# Genetic deficiency of EXOSC10 ribonuclease disrupts spermatogenesis and male fertility in mice

**DOI:** 10.1016/j.jbc.2025.110364

**Published:** 2025-06-11

**Authors:** Junjie Yu, Nana Li, Hong Li, Xiao Wang, Zhengpin Wang

**Affiliations:** Shandong Provincial Key Laboratory of Animal Cell and Developmental Biology, School of Life Sciences, Shandong University, Qingdao, China

**Keywords:** germ cell, infertility, meiosis, ribonuclease, spermatogenesis

## Abstract

Male germ cell meiosis is essential for the generation of haploid spermatozoa in mammals. Oocyte-specific KO of *Exosc10* results in female subfertility by impairing germinal vesicle breakdown and disrupting the transcriptome during oocyte maturation. However, the involvement of EXOSC10 in the regulation of male germ cell meiosis remains poorly understood. This study explores the pivotal role of EXOSC10 ribonuclease in male germ cell meiosis in mice. Conditional deletion of *Exosc10* in premeiotic germ cells impedes spermatogonial differentiation and obstructs meiotic progression in spermatocytes, characterized by abnormal synapsis and defective meiotic recombination. These disruptions lead to heightened apoptosis, defective meiosis, compromised spermatogenesis, and resultant male sterility. Single-cell RNA-Seq analysis further corroborates these meiotic defects, demonstrating that *Exosc10* deficiency disrupts RNA metabolism, misregulates meiotic gene expression, and impairs rRNA processing. Consequently, the meiotic process is compromised, spermatogenesis is disrupted, and male infertility ensues. Collectively, these findings underscore the critical role of EXOSC10 in maintaining a proper transcriptome during spermatocyte meiotic progression, thereby ensuring normal spermatogenesis and male fertility.

Mammalian spermatogenesis is a complex process that involves spermatogonial mitosis, spermatocyte (Scyte) meiosis, and spermiogenesis (1, 2). Spermatogonial stem cells proliferate and differentiate into progenitor and mature spermatogonia (SPG) (3, 4). Type B SPG differentiate into two primary diploid Scytes, each of which undergoes two meiotic divisions to produce four haploid round spermatids (Stids), initiating spermiogenesis (5). During Scyte meiosis, cells undergo DNA replication, homologous chromosome pairing, synapsis, meiotic recombination, and chromosome segregation (6). Meiotic recombination begins with the formation of DNA double-strand breaks (DSBs), followed by the resection of DNA ends and the loading of single-stranded DNA-binding proteins (RPA, SPATA22, and MEIOB). These proteins recruit BRCA2, which facilitates the loading of DMC1 and RAD51 recombinases onto single-stranded DNA to promote DNA strand invasion. The process culminates in second-end capture and the formation of a double Holliday junction, completing the recombination cycle (7, 8, 9, 10). Abnormalities at any stage of meiosis can lead to meiotic errors and male sterility (11, 12, 13).

The RNA exosome complex consists of nine subunits, forming a two-layered barrel-like structure: a core ring comprising six conserved proteins (EXOSC4–9), with three RNA-binding proteins (EXOSC1–3) on top (14). The core complex itself is inactive and lacks catalytic function. Its catalytic activity is conferred through association with one of three ribonucleases—EXOSC10 (RRP6), DIS3 (RRP44), or DIS3L (15). EXOSC10 is positioned at the apex of the exosome core complex, predominantly localized in the nucleus, and enriched within the nucleolus (16). The EXOSC10-associated RNA exosome complex is responsible for the degradation and processing of a broad spectrum of nuclear transcripts (17, 18, 19). Genetic mutations in RNA exosome–related genes have been linked to a variety of human diseases, including myeloma, diarrhea, and neurodegenerative disorders (20, 21, 22). Recent studies have highlighted the role of EXOSC10 in reproductive biology in mice. For instance, an early study observed that EXOSC10 localizes to the periphery of nucleolus precursor bodies in blastomeres, and global deletion of *Exosc10* disrupts the eight-cell embryo/morula transition during mouse embryogenesis (23). Conditional deletion of *Exosc10* in oocytes from the primordial follicle stage onward, using *Gdf9-Cre* mice, results in complete female infertility because of impaired oogenesis, defective oocyte maturation, and depletion of the ovarian reserve (24). Similarly, inactivation of *Exosc10* in oocytes from the primary follicle stage onward, using *Zp3-Cre* mice, leads to delayed germinal vesicle breakdown, oocyte incompetence, and disruption of preimplantation embryo development, resulting in female subfertility (25).

A prior study has demonstrated that EXOSC10 undergoes post-translational regulation in male germ cells and is indispensable for their growth and development. Conditional disruption of *Exosc10* in meiotic germ cells impairs spermatogenesis, reduces sperm count, and causes male subfertility (26). However, the precise mechanisms by which EXOSC10 regulates Scyte meiosis and how EXOSC10-mediated RNA decay influences the transcriptomic landscape of Scytes remain unclear. In the present study, to elucidate the precise function of EXOSC10 in Scyte meiosis, conditional KO (cKO) mice lacking *Exosc10* specifically in germ cells were generated by crossing *Exosc10* Floxed/Floxed (*Exosc10*^*F/F*^) mice with *Stra8-Cre* mice. Our findings, in contrast to previous reports (26), reveal that *Exosc10* cKO males are completely sterile. EXOSC10 loss disrupts spermatogonial differentiation and severely impedes meiotic progression in Scytes, accompanied by elevated apoptosis. This culminates in the absence of postmeiotic germ cells, the failure of spermatogenesis, and a lack of mature sperm, ultimately leading to male infertility. Single-cell RNA-Seq (scRNA-Seq) analysis further demonstrates that *Exosc10* deficiency disrupts RNA degradation, misregulates gene expression, and impairs rRNA processing, contributing to defective spermatogenesis and male sterility. Collectively, the results of this study establish that EXOSC10 plays a critical role in shaping the transcriptome during Scyte meiotic progression, thereby ensuring proper spermatogenesis and male fertility.

## Results

### *Exosc10* expression in the mouse testes

An earlier study has detailed the expression pattern of EXOSC10 during mouse spermatogenesis. To investigate its role in mouse testes, the expression levels of *Exosc10* mRNA across various tissues and developmental stages of mouse testes were assessed using RT–quantitative PCR (RT–qPCR). The results revealed that *Exosc10* mRNA was highly expressed in the testes and exhibited a gradual increase from postnatal day 0 (P0) to P60 (Fig. S1, *A* and *B*). To further explore the localization of EXOSC10 in early testicular germ cells, immunofluorescence analysis was performed on testes from P0, P7, P10, and P15 wildtype mice, staining with antibodies against DEAD box helicase 4 (DDX4) and EXOSC10. Immunostaining demonstrated that EXOSC10 was present in the nuclei of SPG and Scytes (Fig. S1*C*). In addition, costaining of EXOSC10 and synaptonemal complex protein 3 (SYCP3) in adult wildtype testes confirmed that EXOSC10 colocalized with SYCP3 in Scytes (Fig. S1*D*). These results indicate that EXOSC10 is expressed in both SPG and Scytes, thus providing a foundation for conditional ablation of EXOSC10 in meiotic Scytes to elucidate its role in meiosis and spermatogenesis.

### EXOSC10 is indispensable for spermatogenesis and male fertility

To examine the function of EXOSC10 in spermatogenesis, *Exosc10* was conditionally ablated by crossing *Exosc10* Floxed/Floxed (*Exosc10*^*F/F*^) mice with *Stra8-Cre* mice (Fig. S2, *A* and *B*). In these cKO mice, CRE recombinase was first expressed at P3 in a subset of SPG and subsequently in all preleptotene stage Scytes. Confirmation of successful KO in *Stra8-Cre*; *Exosc10 F/−* mice (referred to as *Exosc10* cKO) and their control siblings was achieved through genomic PCR assays (Fig. S2*C*). Moreover, RT–qPCR and Western blot analyses revealed that *Exosc10* mRNA and protein levels were significantly reduced in the P12 *Exosc10* cKO testes compared with control testes (Fig. S2, *D*–*F*). Coexpression analysis of EXOSC10 and SYCP3 in the P12 testes further demonstrated that EXOSC10 signals were absent in SYCP3-positive Scytes of *Exosc10* cKO mice (Fig. S2*G*), confirming the successful depletion of EXOSC10 in Scytes.

**Figure 1:**
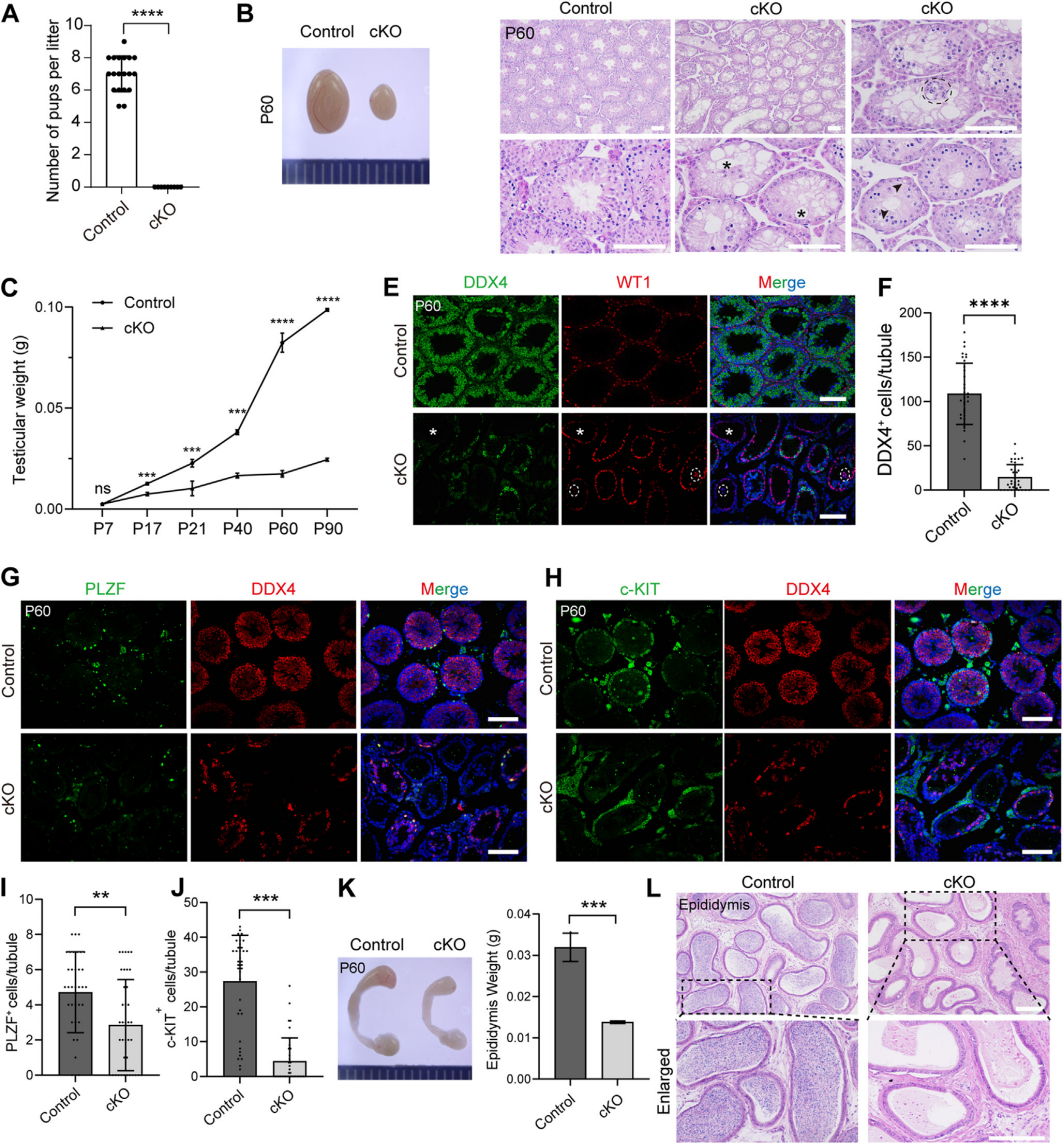
**EXOSC10 is essential for spermatogenesis and male fertility.***A*, fertility assessment of adult control and *Exosc10* cKO male mice. Data presented as the mean number of litters ± SD. ∗∗∗∗*p* < 0.0001. *B*, testicular morphology of P60 control and *Exosc10* cKO mice. *C*, testicular weight measurements at various ages in control and *Exosc10* cKO mice. Data are presented as mean ± SD from three biologically independent testes from three distinct animals; ns, no significance, ∗∗∗*p* < 0.001, ∗∗∗∗*p* < 0.0001. *D*, H&E-stained testicular sections from P60 control and *Exosc10* cKO mice. *Asterisks* denote tubules devoid of spermatogenic cells. *Arrowheads* indicate vacuoles. Scale bar represents 100 μm. *E*, dual-immunofluorescence staining for DDX4 and WT1 in P60 control and *Exosc10* cKO testes. *Asterisks* indicate Sertoli cell–only tubules. *Dashed circles* represent multinucleated giant Sertoli cells. Scale bar represents 100 μm. *F*, quantitative analysis of DDX4-positive cells per tubule in P60 control and *Exosc10* cKO testes. Data presented as the mean ± SD from n = 3 biologically independent experiments. ∗∗∗∗*p* < 0.0001. *G*, coimmunostaining of PLZF and DDX4 in P60 control and *Exosc10* cKO testes. Scale bar represents 100 μm. *H*, coexpression of c-KIT and DDX4 in P60 control and *Exosc10* cKO testes. Scale bar represents 100 μm. *I* and *J*, statistical analyses of PLZF-positive (*I*) and c-KIT-positive (*J*) cells per tubule in P60 control and *Exosc10* cKO testes. Data are presented as mean ± SD from three biologically independent experiments; ∗∗*p* < 0.01, ∗∗∗*p* < 0.001. *K*, epididymal morphology in P60 control and *Exosc10* cKO mice (*left*). Epididymal weight measurements in P60 control and *Exosc10* cKO mice (*right*). Data are presented as mean ± SD from three biologically independent epididymides from three different animals. ∗∗∗*p* < 0.001. *L*, histological analysis of epididymis from P60 control and *Exosc10* cKO mice. *Lower panels* are higher-magnification images of boxed regions shown in *upper panels*, captured from the same biological specimens. Scale bar represents 100 μm. Representative data from n = 3 independent biological replicates (*D*, *E*, *G*, *H*, and *L*) with consistent results across conditions. cKO, conditional KO; DDX4, DEAD box helicase 4; WT1, Wilms tumor 1.

Subsequently, the reproductive phenotype of *Exosc10* cKO male mice was assessed. Over a 6-month mating period, adult *Exosc10* cKO males were found to be completely infertile (Fig. 1*A*). At P60, *Exosc10* cKO males exhibited significantly smaller testes, with testicular weight significantly reduced from P17 to 3 months (Fig. 1, *B* and *C*). Histological examination revealed a range of abnormalities in the P60 *Exosc10* cKO testes (Fig. 1*D*). The majority of seminiferous tubules displayed a marked reduction in spermatogenic cell numbers (indicated by *asterisks*), with some tubules exhibiting multinucleated giant Sertoli cells and numerous small and large vacuoles. To further substantiate these histological observations, dual-immunofluorescence staining for DDX4 (a germ cell–specific marker) and Wilms tumor 1 (WT1, a Sertoli cell–specific marker) was performed (Fig. 1*E*). The coimmunostaining revealed that several mutant seminiferous tubules were entirely devoid of DDX4-positive cells. In addition, WT1-positive Sertoli cells were fused into multinucleated giant cells in certain mutant tubules, whereas most mutant tubules contained a markedly reduced number of spermatogenic cells, consistent with the H&E staining observations. Statistical analysis further confirmed a significant reduction in germ cell numbers in *Exosc10* cKO testes (Fig. 1*F*). Moreover, coimmunostaining of DDX4 with PLZF (ZBTB16, a marker for undifferentiated SPG) and with c-KIT (a marker for differentiated SPG) demonstrated a significant decrease in the numbers of PLZF-positive and c-KIT-positive cells per tubular cross-section in *Exosc10* cKO testes (Fig. 1, *G*–*J*). Correspondingly, epididymal size and weight were significantly reduced in *Exosc10* cKO mice compared with controls (Fig. 1*K*), and no mature spermatozoa were present in the cauda epididymis of *Exosc10* cKO mice (Fig. 1*L*). Collectively, these results establish that EXOSC10 is essential for spermatogenesis, with its deletion leading to substantial germ cell loss, defective spermatogenesis, and male infertility.

### EXOSC10 ablation impairs spermatogonial development

Given that *Stra8*-induced CRE expression commences in premeiotic germ cells, this study initially examined whether spermatogonial development was affected. Testes isolated from P3 control and *Exosc10* cKO mice were analyzed. Histological analysis, coimmunostaining with DDX4 and WT1, coexpression of DDX4 and PLZF, and statistical assessments indicated that *Exosc10* cKO testes exhibited similar histology and comparable numbers of DDX4-positive and PLZF-positive cells to control testes (Fig. S3, *A*–*E*). However, at P7, histological analysis and coimmunostaining of DDX4 and WT1, DDX4 and PLZF, and DDX4 and c-KIT, along with statistical analyses, revealed that the numbers of DDX4-positive, PLZF-positive, and c-KIT-positive cells were significantly reduced in *Exosc10* cKO testes (Fig. S4, *A*–*G*). Western blot analysis further confirmed the substantial decrease in DDX4, PLZF, and c-KIT protein levels in *Exosc10* cKO testes (Fig. S4, *H* and *I*). These results indicate that EXOSC10 ablation causes a notable reduction in the number of SPG.

### EXOSC10 is required for Scyte meiosis

**Figure 2:**
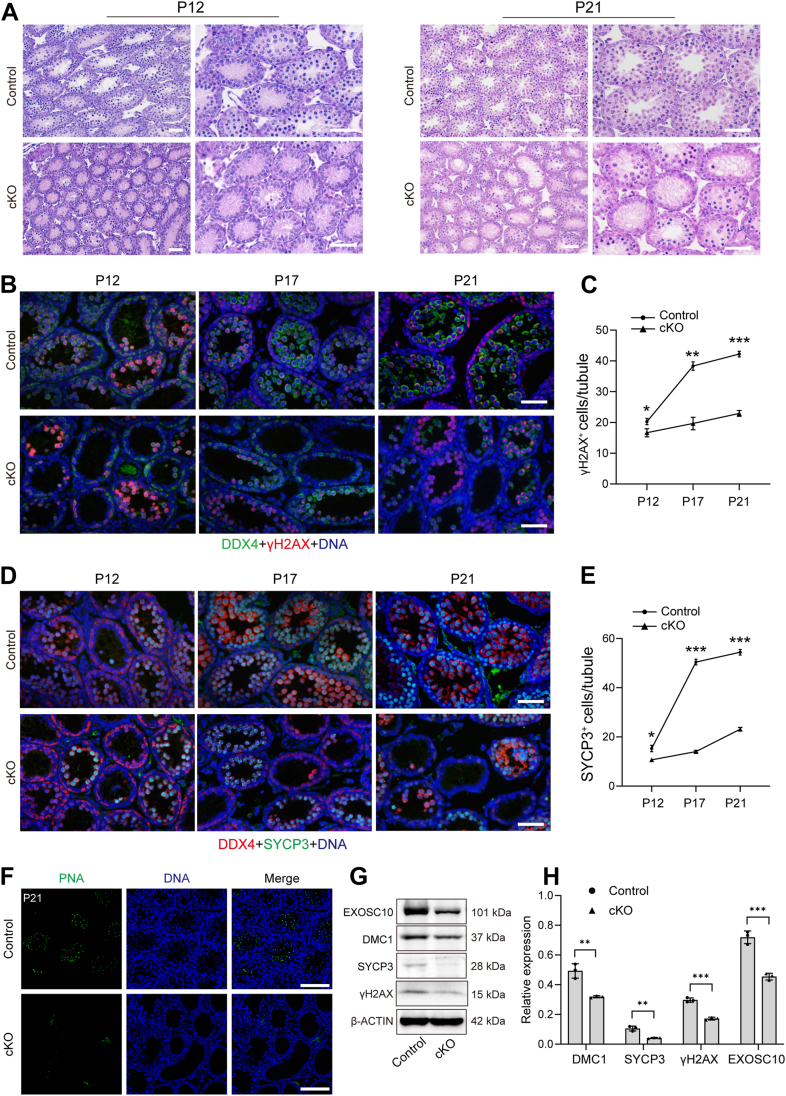
**EXOSC10 is critical for male germ cell meiosis.***A*, H&E staining of testicular sections from control and *Exosc10* cKO mice at P12 and P21. Scale bar represents 100 μm. *B*, dual-immunofluorescence staining for DDX4 and γH2AX in P12, P17, and P21 control and *Exosc10* cKO testes. DNA was stained with Hoechst 33342. Scale bar represents 100 μm. *C*, quantification of γH2AX-positive cells per tubule in control and *Exosc10* cKO mice at P12, P17, and P21. Data are presented as the mean ± SD from three biologically independent experiments; ∗*p* < 0.05, ∗∗*p* < 0.01, ∗∗∗*p* < 0.001. *D*, coexpression of DDX4 and SYCP3 in P12, P17, and P21 control and *Exosc10* cKO testes. Scale bar represents 100 μm. *E*, statistical analysis of SYCP3-positive cells per tubule in control and *Exosc10* cKO mice at P12, P17, and P21. Data are presented as mean ± SD from three biologically independent experiments; ∗*p* < 0.05, ∗∗∗*p* < 0.001. *F*, PNA staining of testicular sections from P21 control and *Exosc10* cKO mice. DNA was stained with Hoechst 33342. Scale bar represents 100 μm. *G*, immunoblot analysis of EXOSC10, DMC1, SYCP3, and γH2AX in P16 control and *Exosc10* cKO testes, with β-ACTIN as a loading control. *H*, relative expression levels of EXOSC10, DMC1, SYCP3, and γH2AX. Data are presented as the mean ± SD from three biologically independent experiments. ∗∗*p* < 0.01, ∗∗∗*p* < 0.001. Representative data from n = 3 independent biological replicates (*A*, *B*, *D*, and *F*) with consistent results across conditions. cKO, conditional KO; DDX4, DEAD box helicase 4; PNA, peanut agglutinin; SYCP3, synaptonemal complex protein 3.

To further investigate whether Scyte meiosis is affected in *Exosc10* cKO testes, testes isolated from P12, P17, and P21 were analyzed. Histological examination of testicular sections from P12 and P21 control and *Exosc10* cKO mice revealed a significant reduction in germ cell numbers within the seminiferous tubules, particularly in the central regions of the tubules (Fig. 2*A*), suggesting a defect in Scyte meiosis. To validate this, the expression patterns of meiotic markers, including SYCP3, a key component of the synaptonemal complex, and γH2AX, a protein involved in DSB repair, were examined. Coimmunostaining with DDX4 and γH2AX antibodies, DDX4 and SYCP3 antibodies, along with statistical analysis in P12, P17, and P21 control and *Exosc10* cKO testes, revealed a significant reduction in the number of γH2AX-positive and SYCP3-positive Scytes during meiosis in *Exosc10* cKO testes (Fig. 2, *B*–*E*). In addition, TUNEL assays demonstrated a marked increase in apoptotic signals in *Exosc10* cKO testes at both P12 and P21 (Fig. S5, *A*–*D*). The observed increase in apoptosis was further supported by immunoblotting analysis of cleaved Caspase-3, showing significantly higher levels in testes of P12 and P16 *Exosc10* cKO mice compared with controls (Fig. S5, *E* and *F*). Further examination of testicular sections from P21 control and *Exosc10* cKO mice, stained with FITC-labeled peanut agglutinin (PNA), which marks the acrosome of round Stids, showed a complete absence of PNA signals in *Exosc10* cKO testes, indicating the lack of round Stids in these testes (Fig. 2*F*). Immunoblotting confirmed that the expression levels of meiosis-related proteins, DMC1, γH2AX, and SYCP3, were significantly reduced in P16 *Exosc10* cKO testes (Fig. 2, *G* and *H*). Collectively, these results demonstrate that conditional deletion of EXOSC10 leads to severe Scyte loss, underscoring the critical role of EXOSC10 in normal Scyte meiosis and survival.

### EXOSC10 is essential for Scyte meiotic progression

**Figure 3:**
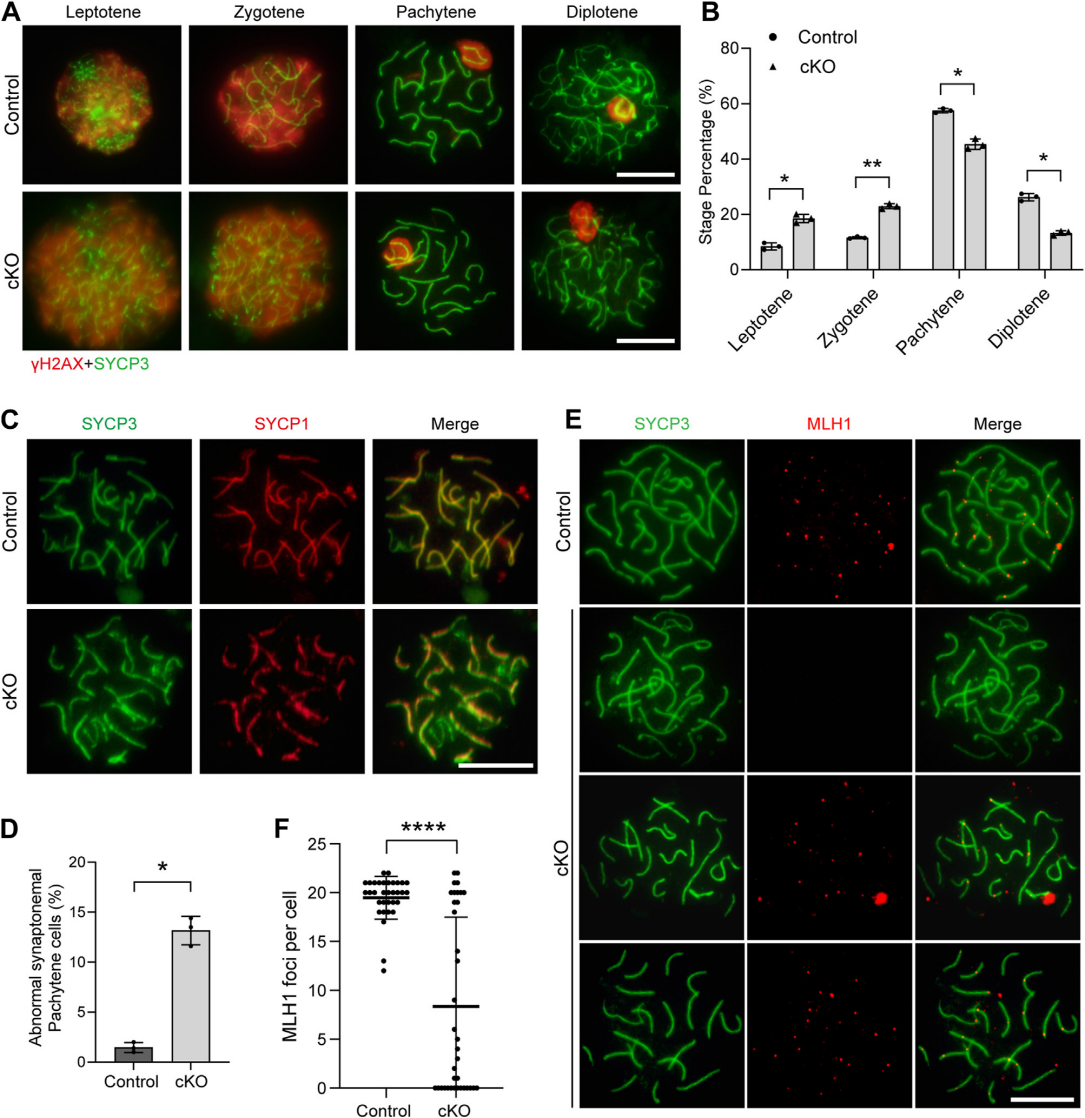
***Exosc10* depletion impairs meiotic progression.***A*, chromosome spreads of spermatocytes (Scytes) from P21 control and *Exosc10* cKO mice, coimmunostained with γH2AX and SYCP3 antibodies. Scale bar represents 10 μm. *B*, quantification of Scyte stages (leptotene, zygotene, pachytene, and diplotene) in the testes of control and *Exosc10* cKO mice. Data presented as mean ± SD from three biologically independent experiments; ∗*p* < 0.05, ∗∗*p* < 0.01. *C*, coimmunostaining of SYCP1 and SYCP3 in chromosome spreads of Scytes from P16 control and *Exosc10* cKO testes. Scale bar represents 10 μm. *D*, quantification of abnormal synaptonemal Scytes. Data expressed as mean ± SD from n = 3 biologically independent experiments; ∗*p* < 0.05. *E*, chromosome spreads of Scytes from P16 control and *Exosc10* cKO testes, stained with SYCP3 and MLH1 antibodies. Scale bar represents 10 μm. *F*, quantification of MLH1 foci in P16 control and *Exosc10* cKO Scytes. Data presented as mean ± SD from three biologically independent experiments; ∗∗∗∗*p* < 0.0001. cKO, conditional KO; SYCP3, synaptonemal complex protein 3.

Primary Scytes undergo progression through the leptotene (P10), zygotene (P12), pachytene (P14), and diplotene (P17) stages, culminating in haploid Stid formation by approximately P21. To elucidate the meiotic impairment in *Exosc10* cKO testes, chromosome spreads from Scytes isolated from P21 control and *Exosc10* cKO testes were subjected to staining with SYCP3 and γH2AX antibodies. In control testes, the distribution of Scytes across the leptotene, zygotene, pachytene, and diplotene stages was approximately 7.4%, 9.0%, 57.5%, and 26.1%, respectively (Fig. 3, *A* and *B*). In contrast, the *Exosc10* cKO testes exhibited 18.5%, 22.9%, 45.6%, and 13.0% of Scytes at these stages, respectively (Fig. 3, *A* and *B*), indicative of disrupted meiotic progression. To explore the underlying cause of this meiotic deficiency, chromosome synapsis was examined in *Exosc10* cKO testes. Scyte chromosome spreads were costained with SYCP3 and SYCP1, revealing near-complete colocalization of SYCP3 and SYCP1 on autosomes in control pachytene Scytes. However, *Exosc10* cKO testes exhibited a notable increase in the proportion of pachytene Scytes with disrupted SYCP1 signals on autosomes, suggesting a synapsis defect following EXOSC10 depletion (Fig. 3, *C* and *D*). Given the presence of a small subset of aberrantly synapsed pachytene Scytes, meiotic DSB repair was further assessed using DMC1 and SYCP3 immunostaining. At the zygotene stage, DMC1 foci were comparable between control and *Exosc10* cKO Scytes. However, a significant increase in DMC1 foci was observed in *Exosc10* cKO Scytes at the pachytene stage (Fig. S6, *A* and *B*), indicative of a defect in DSB repair. At the pachytene stage, MutL homolog 1 (MLH1) foci, marking successful crossover during DNA recombination, were present in control Scytes, whereas their numbers were significantly reduced in some pachytene Scytes of *Exosc10* cKO testes (Fig. 3, *E* and *F*), suggesting a crossover defect. These results collectively demonstrate that EXOSC10 depletion disrupts meiotic progression in Scytes, highlighting its critical role in ensuring proper meiotic development.

### scRNA-Seq analysis of *Exosc10* cKO testes

**Figure 4:**
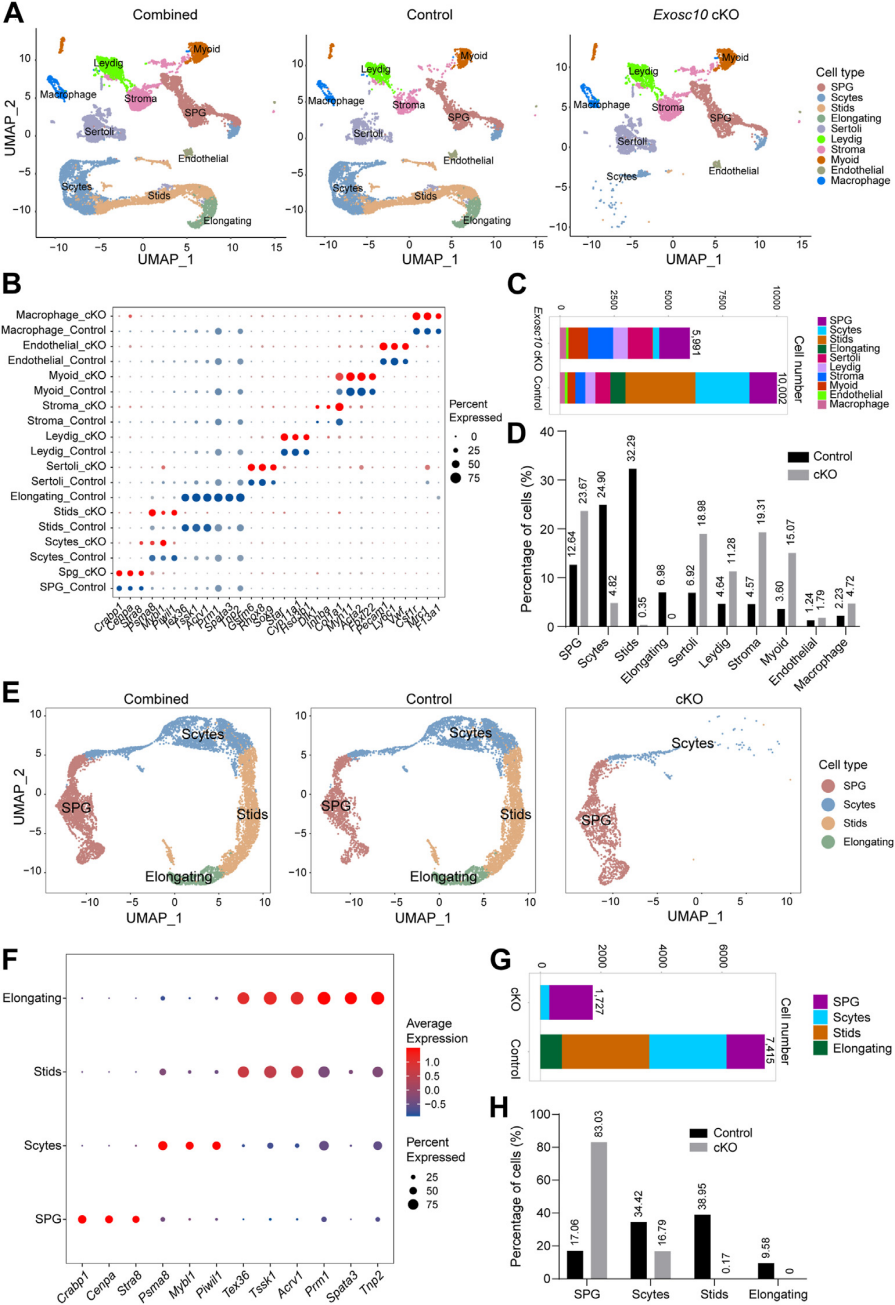
**scRNA-Seq analysis of P21 control and *Exosc10* cKO testes.***A*, UMAP plots of combined (*left*), control (*middle*), and *Exosc10* cKO (*right*) testicular cells. Each *dot* represents a single cell, with clusters identified by distinct colors. Cell populations include spermatogonia (SPG), spermatocytes (Scytes), round spermatids (Stids), elongating Stids (Elongating), Sertoli cells, Leydig cells, stromal cells, myoid cells, endothelial cells, and macrophages. *B*, dot plot showing expression levels of selected marker genes in each cluster. *C*, bar graph illustrating the number of cells in each cluster from control and *Exosc10* cKO testes. *D*, bar graph showing the percentage distribution of cell clusters in control and *Exosc10* cKO testes. *E*, UMAP visualization of combined (*left*), control (*middle*), and *Exosc10* cKO (*right*) germ cells, with each *dot* representing a single germ cell and distinct germ cell clusters represented by different colors. *F*, dot plot for the expression of selected marker genes in each germ cell cluster. *G*, bar graph showing the number of germ cells in each cluster from control and *Exosc10* cKO testes. *H*, bar graph displaying the percentage distribution of germ cell clusters in control and *Exosc10* cKO testes. cKO, conditional KO; scRNA-Seq, single-cell RNA-Seq; UMAP, Uniform Manifold Approximation and Projection.

To investigate the cellular composition and transcriptomic alterations in *Exosc10* cKO testes, testicular cells were isolated from P21 control and *Exosc10* cKO testes, followed by scRNA-Seq analysis using the 10X Genomics platform. After quality control, 10,002 control and 5991 *Exosc10* cKO testicular cells were retained for clustering analysis. These cells were classified into four germ cell subtypes—SPG, Scyte, round Stids), and early elongating Stids (Elongating)—as well as six somatic cell populations, including endothelial cells, macrophages, myoid cells, Leydig cells, stromal cells, and Sertoli cells, based on Uniform Manifold Approximation and Projection (UMAP) and marker-gene analyses (Fig. 4, *A* and *B*). Notably, statistical analysis revealed a significant reduction in the proportions of Scytes (4.82% *versus* 24.90%), round Stids (0.35% *versus* 32.29%), and early elongating Stids (0.00% *versus* 6.98%) in *Exosc10* cKO testes compared with controls (Fig. 4, *C* and *D*), further indicating that conditional deletion of EXOSC10 in premeiotic germ cells results in a marked loss of meiotic Scytes and postmeiotic germ cells.

To further characterize the defects in germ cell development, all germ cells were extracted for reclustering. UMAP and marker-gene analysis identified four distinct clusters corresponding to SPG, Scytes, round Stids, and early elongating Stids (Fig. 4, *E* and *F*). Statistical analysis revealed a substantial increase in the proportion of SPG (83.03% *versus* 17.06%), whereas the percentages of Scytes (16.79% *versus* 34.42%), round Stids (0.17% *versus* 38.95%), and early elongating Stids (0.00% *versus* 9.58%) were significantly decreased in *Exosc10* cKO testes compared with control testes (Fig. 4, *G* and *H*). These results further support the presence of abnormal meiosis, characterized by a reduced proportion of Scytes and the complete absence of postmeiotic germ cells upon EXOSC10 deletion. In summary, scRNA-Seq analysis demonstrates that EXOSC10 ablation leads to a significant decline in the number and proportion of meiotic Scytes and postmeiotic germ cells, implicating a disruption of meiotic progression in the absence of EXOSC10.

### scRNA-Seq analysis of Scyte population abundance in *Exosc10* cKO testes

**Figure 5:**
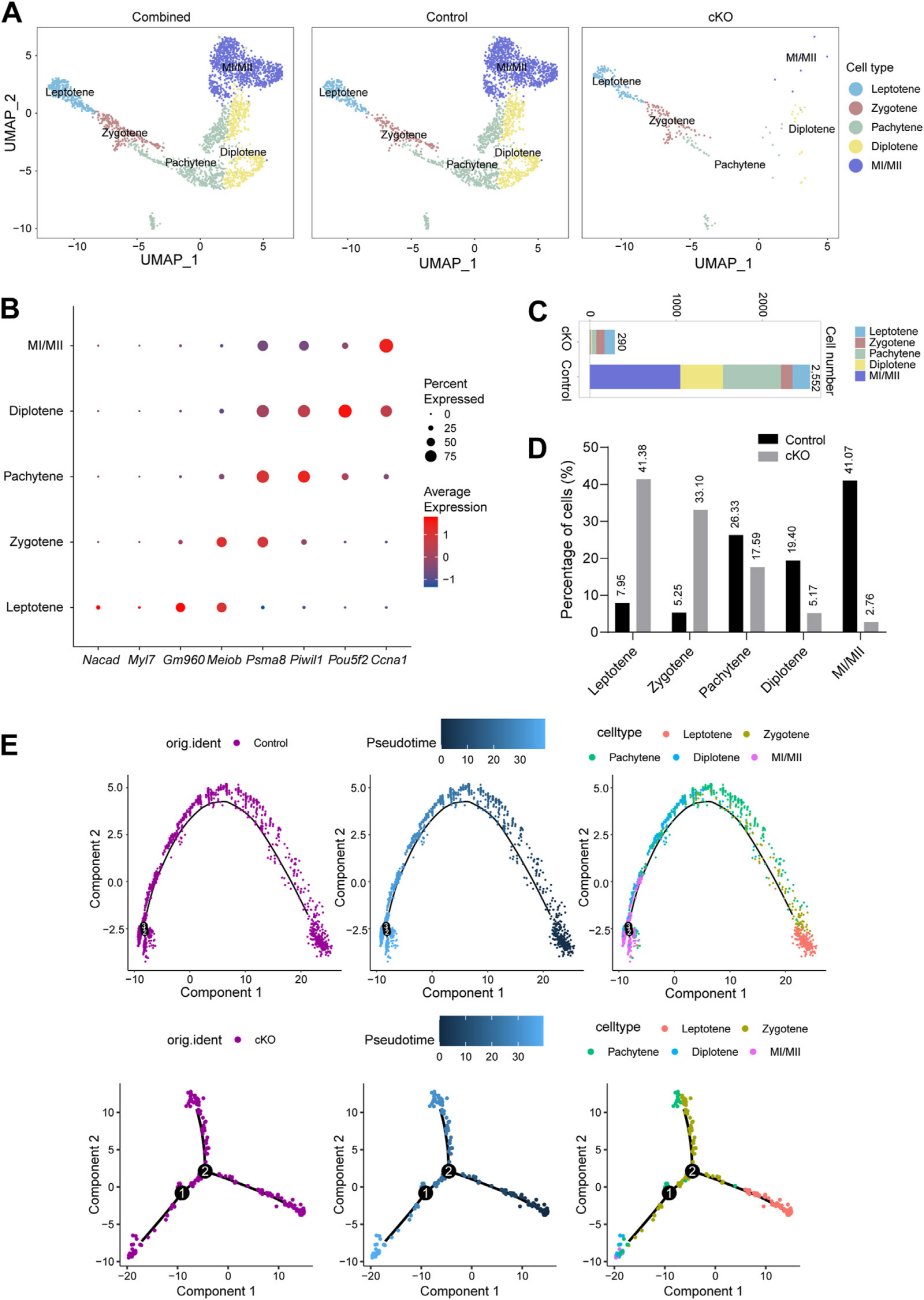
**scRNA-Seq analysis of control and *Exosc10* cKO meiotic spermatocytes (Scytes).***A*, UMAP visualization of combined (*left*), control (*middle*), and *Exosc10* cKO (*right*) Scyte populations. *B*, dot plot displaying the expression of selected marker genes across different cell clusters. *C*, bar graph depicting the number of cells in various subtypes of Scytes from control and *Exosc10* cKO testes. *D*, bar graph showing the percentage distribution of Scyte subtypes in control and *Exosc10* cKO testes. *E*, pseudotime trajectory analysis of meiotic Scytes from control and *Exosc10* cKO mice. cKO, conditional KO; scRNA-Seq, single-cell RNA-Seq; UMAP, Uniform Manifold Approximation and Projection.

To further elucidate the role of EXOSC10 in meiotic progression, meiotic Scytes were extracted for reclustering. Following quality control, 2552 control cells and 290 *Exosc10* cKO cells were analyzed. UMAP and marker-gene analyses identified five distinct subtypes based on the expression patterns of meiosis-related marker genes: leptotene Scytes (*Nacad*, *Myl7*, and G*m*960), zygotene Scytes (*Meiob*), pachytene Scytes (*Psma8* and *Piwil1*), diplotene Scytes (*Pou5f2*), and MI/MII Scytes (*Ccna1*) (Fig. 5, *A* and *B*). Statistical analysis revealed that in control testes, 7.95%, 5.25%, 26.33%, 19.40%, and 41.07% of cells were sorted into the leptotene, zygotene, pachytene, diplotene, and MI/MII subtypes, respectively, whereas in *Exosc10* cKO testes, 41.38%, 33.10%, 17.59%, 5.17%, and 2.76% of cells were allocated to these stages, respectively (Fig. 5, *C* and *D*). A significant increase in the proportion of leptotene and zygotene Scytes and a notable decrease in pachytene, diplotene, and MI/MII Scytes were observed in *Exosc10* cKO testes, indicating a severe disruption in Scyte meiotic progression because of the absence of EXOSC10. Pseudotime analysis traced the developmental trajectory of Scytes from the leptotene to the MI/MII stage in control testes. However, in *Exosc10* cKO testes, this trajectory was disturbed, exhibiting branching points, with some Scytes prematurely halting at the pachytene stage (Fig. 5*E*). These results indicate that EXOSC10 is essential for Scyte meiotic progression, and that *Exosc10* deficiency disrupts this process, resulting in a significant meiotic arrest at the pachytene stage.

### Transcriptome signatures of *Exosc10* cKO meiotic Scytes

**Figure 6:**
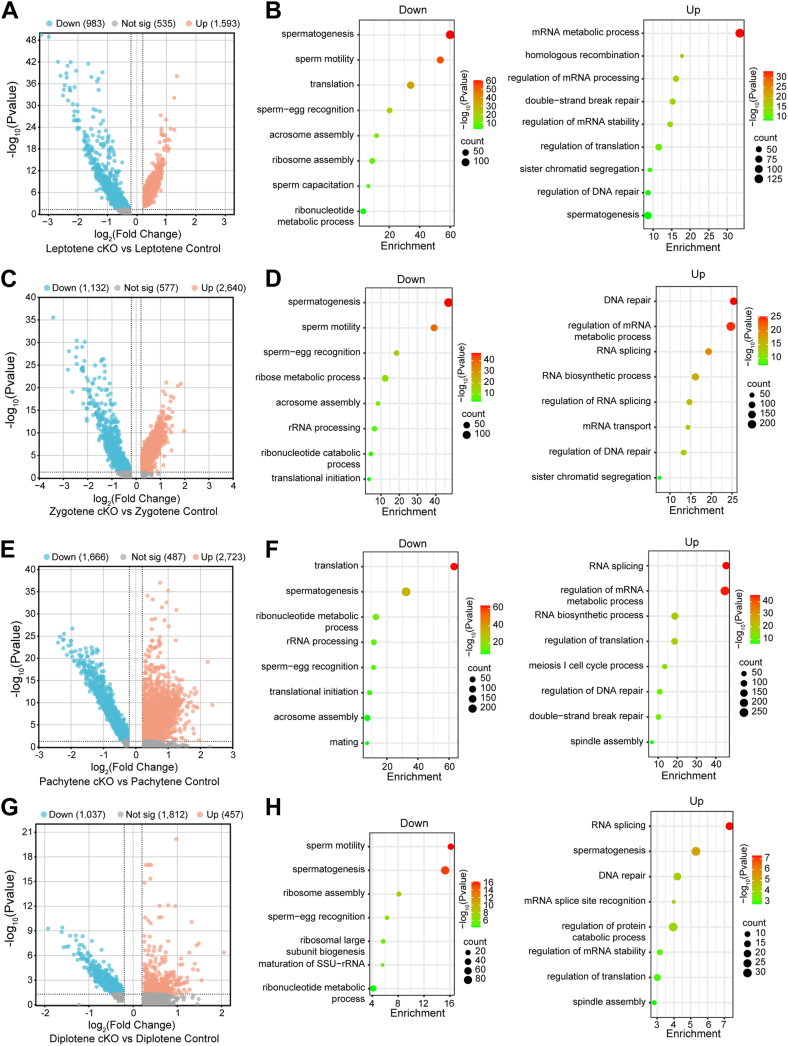
**Transcriptome analysis of control and *Exosc10* cKO meiotic spermatocytes (Scytes).***A*, Volcano plot of DEGs in *Exosc10* cKO leptotene Scytes, with a significance threshold of *p* < 0.05 and a |log_2_ fold change| >0.2. *B*, dot plots of Gene Ontology (GO) terms for downregulated (*left*) and upregulated (*right*) transcripts in *Exosc10* cKO leptotene Scytes, based on Metascape analysis. *C*, same as (*A*), but for *Exosc10* cKO zygotene Scytes. *D*, same as (*B*), but showing GO terms for downregulated and upregulated transcripts in *Exosc10* cKO zygotene Scytes. *E*, same as (*A*), but for *Exosc10* cKO pachytene Scytes. *F*, same as (*B*), but showing GO terms for downregulated and upregulated transcripts in *Exosc10* cKO pachytene Scytes. *G*, same as (*A*) but for *Exosc10* cKO diplotene Scytes. *H*, same as (*B*) but showing GO terms for downregulated and upregulated transcripts in *Exosc10* cKO diplotene Scytes. cKO, conditional KO; DEG, differentially expressed gene.

To investigate the underlying molecular mechanisms, differential expression and Gene Ontology (GO) enrichment analyses were performed on all four Scyte subtypes from the scRNA-Seq data. With a cutoff of *p* < 0.05 and |log_2_ fold change| >0.2, 2576 differentially expressed genes (DEGs) (1593 upregulated and 983 downregulated) were identified in *Exosc10* cKO leptotene Scytes, 3772 DEGs (2640 upregulated and 1132 downregulated) in zygotene Scytes, 4389 DEGs (2723 upregulated and 1666 downregulated) in pachytene Scytes, and 1494 DEGs (457 upregulated and 1037 downregulated) in diplotene Scytes (Fig. 6, *A*, *C*, *E* and *G*). Notably, a substantial number of genes were dysregulated in leptotene-, zygotene-, and pachytene-stage Scytes following *Exosc10* deletion, with upregulated genes outnumbering downregulated ones, suggesting defects in RNA degradation processes. GO enrichment analysis of DEGs revealed that downregulated transcripts were primarily enriched in processes related to spermatogenesis, rRNA processing, ribosome assembly, and translation, whereas upregulated genes were predominantly involved in spermatogenesis, DNA repair, mRNA metabolism, and regulation of translation (Fig. 6, *B*, *D*, *F* and *H*).

**Figure 7:**
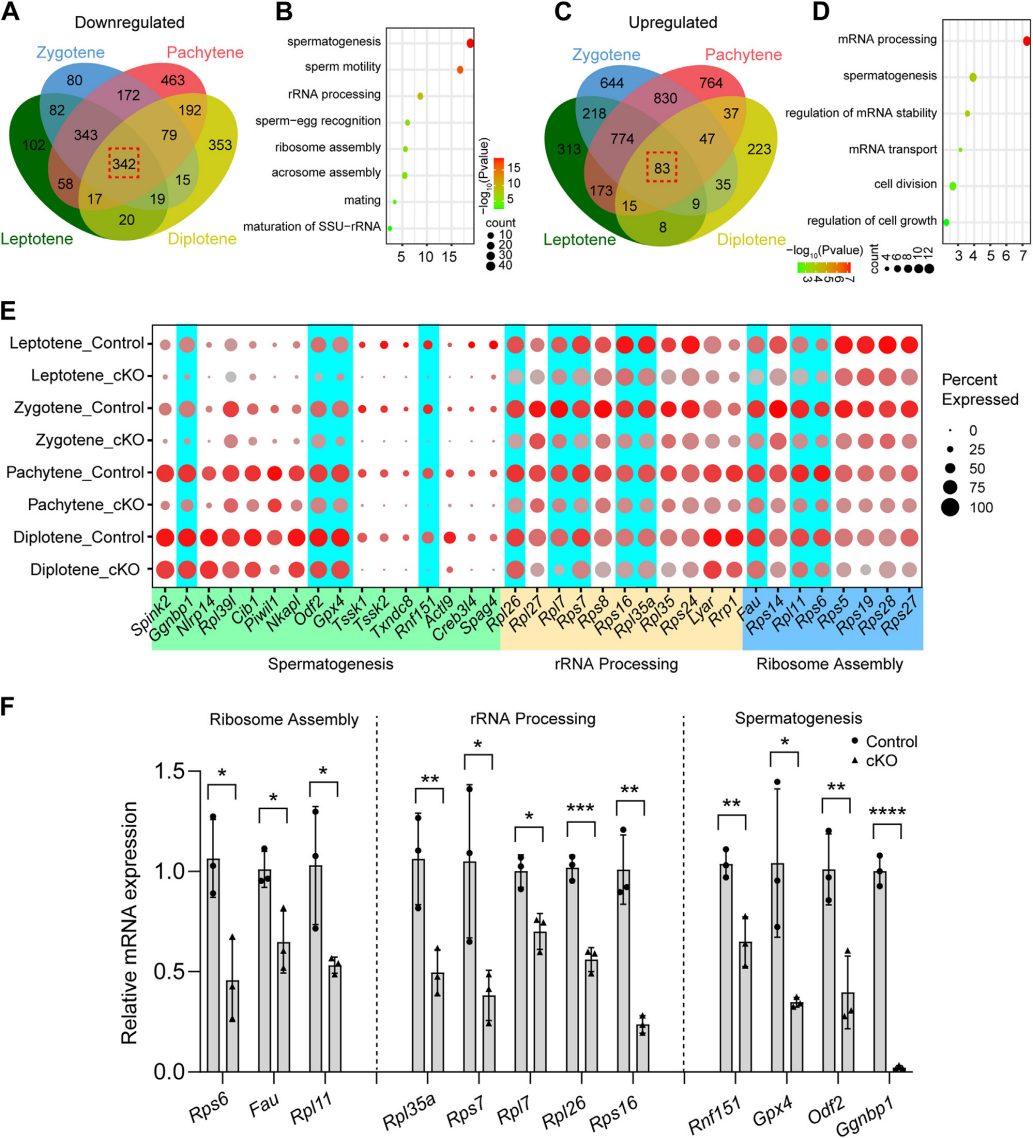
**Combined analysis of transcript changes in *Exosc10* cKO spermatocytes (**Scytes**) during meiotic prophase I.***A*, Venn diagram illustrating downregulated genes across leptotene, zygotene, pachytene, and diplotene Scytes between control and *Exosc10* cKO samples. *B*, dot plot showing the Gene Ontology (GO) terms for overlapping downregulated genes in (*A*). *C*, same as (*A*) but for upregulated genes. *D*, dot plot showing the GO terms for overlapping upregulated genes in (*C*). *E*, dot plot displaying selected downregulated genes associated with spermatogenesis, rRNA processing, and ribosome assembly. *F*, RT–qPCR analysis of expression levels of genes linked to ribosome assembly, rRNA processing, and spermatogenesis in isolated spermatogenic cells from control and *Exosc10* cKO mice. The expression level of genes in control relative to *β-actin* was set to 1. Data are presented as mean ± SD from three biologically independent replicates. ∗*p* < 0.05, ∗∗*p* < 0.01, ∗∗∗*p* < 0.001, and ∗∗∗∗*p* < 0.0001. cKO, conditional KO; qPCR, quantitative PCR.

The DEGs across leptotene, zygotene, pachytene, and diplotene Scytes were subjected to overlap analysis. A significant overlap of downregulated genes was observed among all four stages, with 342 genes common to each stage (Fig. 7*A*). These 342 overlapping genes were markedly enriched in GO terms related to spermatogenesis, rRNA processing, and ribosome assembly (Fig. 7*B*). Likewise, 83 upregulated genes were found to overlap across all four stages (Fig. 7*C*), predominantly involved in spermatogenesis and mRNA processing (Fig. 7*D*). A subset of downregulated genes linked to rRNA processing, ribosome assembly, and spermatogenesis is highlighted in Figure 7*E*, whereas a group of upregulated genes associated with mRNA processing, mRNA stability regulation, spermatogenesis, cell division, and cell growth control is presented in Figure S8*A*. To validate these transcriptomic changes in Scytes, spermatogenic cells were enriched from P12 control and *Exosc10* cKO testes, as described in the Experimental procedures section. Coimmunostaining for DDX4 and WT1 on the isolated spermatogenic cells revealed that approximately 80% of the cells were DDX4 positive in both control and *Exosc10* cKO samples (Fig. S7, *A* and *B*). RT–qPCR analysis confirmed the highest expression of the germ cell marker gene *Ddx4* in these isolated cells (Fig. S7, *C* and *D*). Coexpression of DDX4 and SYCP3 showed that around 70% and 60% of the cells were SYCP3 positive in control and *Exosc10* cKO samples, respectively (Fig. S7, *E* and *F*), indicating that these cells were predominantly Scytes. Further RT–qPCR analysis of isolated spermatogenic cells confirmed a marked reduction in genes associated with ribosome assembly (*e.g.*, *Rps6*, *Fau*, *Rpl11*), rRNA processing (*e.g.*, *Rpl35a*, *Rps7*, *Rpl7*, *Rpl26*, *Rps16*), and spermatogenesis (*e.g.*, *Rnf151*, *Gpx4*, *Odf2*, *Ggnbp1*), alongside a significant increase in genes linked to mRNA processing (*e.g.*, *Srsf10*, *Rnpc3*, *Cdk13*, *Zrsr2*), spermatogenesis (*e.g.*, *Ythdc1*, *Arid4b*, *Sirt1*), and cell division (*e.g.*, *Arf1*, *Ino80*, *Ect2*) in *Exosc10* cKO cells (Figs. 7*F* and S8*B*). In addition, RT–qPCR analysis of meiotic genes in these isolated cells revealed significant dysregulation of several meiotic genes (*e.g.*, *Spo11*, *Rad51*, *Stra8*, *Syce2*, *Rec8*, *Dmc1*, *Sycp1*) in *Exosc10* cKO spermatogenic cells (Fig. S9). In summary, these results strongly suggest that *Exosc10* deficiency in Scytes leads to profound transcriptomic dysregulation, with alterations in genes involved in spermatogenesis, rRNA processing, and ribosome assembly likely contributing to defects in Scyte meiotic progression.

### EXOSC10 ablation leads to pre-rRNA processing defects in spermatogenic cells

**Figure 8:**
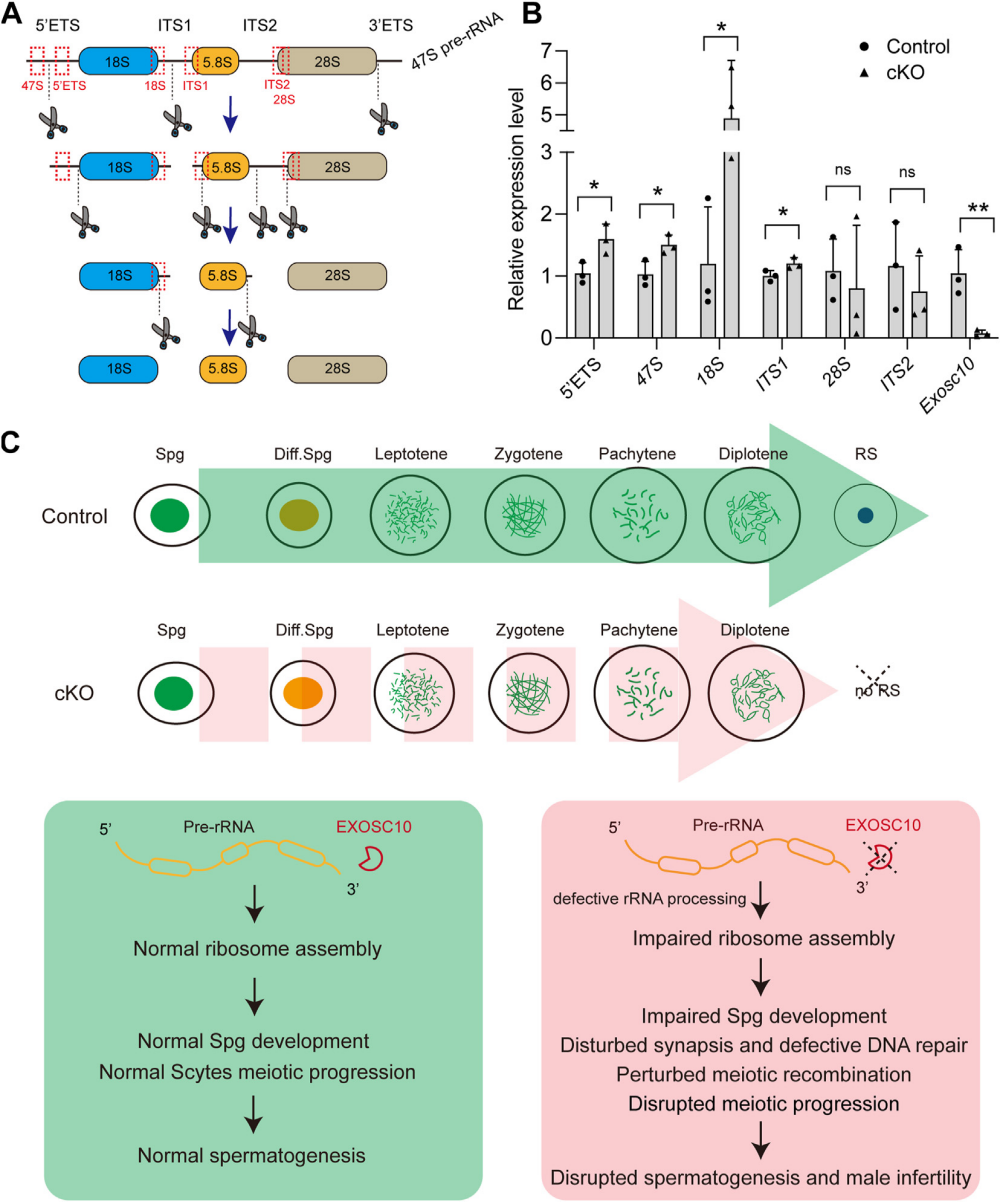
***Exosc10* cKO spermatogenic cells exhibit defective rRNA processing.***A*, schematic representation of the pre-rRNA processing pathway. *B*, RT–qPCR analysis of the expression levels of indicated rRNA precursors in isolated spermatogenic cells from control and *Exosc10* cKO mice. The expression level of genes in control relative to *β-actin* was set to 1. Data are presented as mean ±SD from three biologically independent replicates. ns, no significance, ∗*p* < 0.05, ∗∗*p* < 0.01. *C*, model depicting the role of EXOSC10 in driving spermatogonial development and meiotic progression in spermatocytes. The absence of EXOSC10 results in defective rRNA processing, which may impair ribosome assembly, disrupt spermatogonial development, and hinder meiotic progression, culminating in disrupted spermatogenesis and male sterility. cKO, conditional KO; qPCR, quantitative PCR.

Following transcription from rDNA loci within the nucleoli, 47S pre-rRNA undergoes digestion into 45S rRNA, which is subsequently processed to produce mature rRNAs, including 18S rRNA, 5.8S rRNA, and 28S rRNA (Fig. 8*A*). Given the role of EXOSC10 in mediating 3′ precursor rRNA processing, the expression levels of different regions of the pre-rRNA were examined using various primer sets (Fig. 8*A*). RT–qPCR analysis of isolated spermatogenic cells revealed significant upregulation of the 5′ ETS (including 47S, 5′ ETS) and ITS1 (including 18S, ITS1) regions, whereas the expression level of the ITS2 (including ITS2, 28S) region remained unchanged in *Exosc10* cKO spermatogenic cells (Fig. 8*B*). This suggests that EXOSC10 depletion disrupts pre-rRNA processing in spermatogenic cells, a finding consistent with previous observations in *Exosc10*-deficient oocytes, where increased levels of the 5′ETS and ITS1 regions and defective 3′ end processing of pre-rRNA were observed (25). Collectively, our findings demonstrate that EXOSC10 is essential for normal spermatogonial development and meiotic progression of Scytes during spermatogenesis. Inactivation of *Exosc10* causes defective pre-rRNA processing and potential impairment of ribosome assembly, consequently leading to disruptions in spermatogonial development, synapsis, DNA repair, meiotic recombination, and meiotic progression. These defects culminate in spermatogenesis failure and male infertility (Fig. 8*C*).

## Discussion

An early study documents that inactivation of *Exosc10* in male germ cells, achieved through the use of a *Stra8-Cre* mouse line (27), results in a significant reduction in testicular size and weight, as well as a marked decrease in the number of mature sperm in the epididymis. Although the absence of EXOSC10 severely disrupts spermatogenesis, it does not entirely abolish it, leading to subfertility in the mutant males (26). In contrast to this prior study, our current research, employing a distinct *Stra8-Cre* mouse line (28), reveals a divergent phenotype. Specifically, conditional disruption of EXOSC10 in male germ cells leads to severe disruption of Scyte meiotic progression, culminating in the absence of postmeiotic Stids, failure of subsequent spermatogenesis, and male sterility. Our findings underscore the critical role of EXOSC10-mediated RNA metabolism in maintaining the proper transcriptome of mouse Scytes, ensuring normal meiotic progression, spermatogenesis, and male fertility.

The *Stra8-Cre* mouse line utilized in the earlier study is the STOCK Tg(*Stra8-iCre*)1Reb/J from The Jackson Laboratory, a transgenic mouse model. In that study, the *Stra8-Cre* mouse line was backcrossed with the C57BL/6NRj strain before generating the *Exosc10* cKO mouse lines. In contrast, the *Exosc10*^*F/F*^ mouse line used in our study is C57BL/6JCya, constructed by Cyagen. The *Stra8-Cre* line in our study is a *Stra8-GFPCre* knock-in line, originally maintained on the C57BL/6J background, with the GFPCRE protein inserted into the final coding exon of *Stra8* to enable coexpression of both genes. In addition, while the two LoxP sites flank exon 3 in the earlier study, they flank exons 4 to 6 in our current investigation. Overall, the observed phenotypes may stem from variations in the strategies employed to generate the *Exosc10*^*F/F*^ and *Stra8-Cre* mouse lines, as well as differences in the genetic background of the mouse strains. Nevertheless, both studies conclusively demonstrate that conditional ablation of EXOSC10 disrupts spermatogenesis and impairs male fertility, highlighting its vital role in shaping the transcriptome during meiotic progression in mouse Scytes.

Previous research provides a comprehensive analysis of EXOSC10 expression during meiotic and postmeiotic germ cell differentiation. EXOSC10 is predominantly expressed in the nucleoli of PLZF-positive SPG and pachytene/diplotene Scytes, with moderate expression in the nucleoli of zygotene Scytes and the cytoplasm of metaphase Scytes during meiosis I. Residual signals are confined to one or two foci in secondary Scytes and postmeiotic round Stids, with no detectable signals in elongating Stids or sperm heads (26). These dynamic patterns of EXOSC10 localization further reinforce its critical role in Scyte meiotic progression.

EXOSC10 has been implicated in the regulation of mRNA turnover (17, 29, 30), degradation of long noncoding and enhancer RNAs (18, 31), as well as the 3′ maturation of 5.8S rRNA (32) and pre-rRNA precursor processing (31). Depletion of EXOSC10 in growing oocytes, using *Zp3-Cre* mice, disrupts both poly(A) and non–poly(A) RNA profiles and impairs pre-rRNA processing, thereby hindering the transition from growth to maturation in mouse oocytes. *Exosc10*-null oocytes exhibit severe defects in meiotic resumption and compromised preimplantation embryo development (25). Furthermore, inactivation of EXOSC10 in primordial follicle stage oocytes *via Gdf9-Cre* mice induces premature ovarian insufficiency and significant alterations in the protein profiles associated with RNA processing, meiotic cell cycle progression, and oocyte maturation, suggesting a failure in translational regulation (24). These results collectively suggest that EXOSC10 plays a critical role in regulating oocyte meiotic division through post-transcriptional and translational mechanisms. The present study demonstrates that EXOSC10 also contributes to Scyte meiosis through post-transcriptional regulation. Disruption of RNA degradation in *Exosc10*-null Scytes results in impaired meiotic progression, accompanied by increased cell apoptosis. Together with previous studies, these results emphasize the pivotal role of EXOSC10 ribonuclease in maintaining the transcriptome integrity necessary for the meiotic progression of both Scytes and oocytes, which is essential for fertility.

In a recent report, DIS3L2, an RNA exosome–independent ribonuclease, was identified as a key factor in shaping the transcriptome during mouse spermatogonial differentiation and Scyte meiotic progression. *Dis3l2* deficiency leads to disrupted RNA metabolism, downregulation of cell cycle and spermatogonial differentiation genes, and perturbation of spermatogonial differentiation. In Scytes, *Dis3l2* inactivation results in abnormal RNA processing, impaired translation, and dysregulated meiotic genes, all of which hinder Scyte meiotic progression (33). *Dis3l2* cKO oocytes fail to resume meiosis, arrest at the germinal vesicle stage, and render mutant female mice completely infertile (34). Depletion of DIS3L2 causes the accumulation of uridylated-poly(A) RNA, which exhibits reduced translation in mutant oocytes. These observations further support the critical role of ribonuclease-mediated RNA metabolism in gametogenesis.

To assess the impact of EXOSC10-mediated RNA degradation on the cellular composition and transcriptomic changes in Scytes, analyses were performed on scRNA-Seq data from P21 control and *Exosc10* cKO testes. Cell clustering and pseudotime analyses revealed a predominant presence of leptotene and zygotene Scytes in *Exosc10* cKO testes, with their transition to the pachytene stage and beyond severely disrupted. This finding further underscores the essential role of EXOSC10 in Scyte meiotic progression. Differential expression analysis across leptotene, zygotene, pachytene, and diplotene Scytes provided compelling evidence that RNA degradation disruptions lead to altered RNA abundance. Transcriptome analysis indicates that *Exosc10* deficiency causes substantial dysregulation of Scyte transcripts, disrupting rRNA processing, ribosome assembly, translation, and the expression of genes related to meiosis and spermatogenesis. These disturbances likely underlie the impaired meiotic progression and compromised spermatogenesis observed. RT–qPCR analyses further validated the transcriptomic dysregulation in isolated spermatogenic cells. Collectively, the disrupted genes are likely indirect targets of EXOSC10, with these alterations culminating in the observed defects in meiosis and spermatogenesis.

In our study, 1) EXOSC10 deletion in Scytes (leptotene/zygotene/pachytene stages) induces RNA degradation defects, with upregulated transcripts (enriched in mRNA metabolism, DNA repair, sister chromatid segregation, translation regulation, and spermatogenesis) outnumbering downregulated ones (enriched in rRNA processing, ribosome assembly, translation, and spermatogenesis). 2) EXOSC10 deficiency disrupts meiotic processes (synapsis, DNA repair, and recombination), compromises rRNA maturation (*e.g.*, defective 5′ ETS/ITS1 processing), and impairs ribosome biogenesis (evidenced by downregulation of *Rps6*, *Rps7*, *Rps16*, *Rpl11*, and *Rpl26*). These findings demonstrate that EXOSC10 plays dual roles in 1) shaping the transcriptome to maintain RNA metabolic equilibrium and 2) ensuring rRNA processing/ribosome function for translation regulation during Scyte meiosis. Collectively, EXOSC10 safeguards spermatogenesis by coupling RNA degradation fidelity (preventing aberrant RNA accumulation) with translational competence (*via* ribosome homeostasis), thereby ensuring normal meiotic progression, spermatogenesis, and male fertility. These data establish EXOSC10 as a key regulator of male germ cell differentiation and meiosis, modulating the transcriptome during these critical developmental stages that are essential for spermatogenesis and male fertility.

## Experimental procedures

### Animals

All animal procedures were conducted following the guidelines approved by the Animal Research Ethics Committee of the School of Life Sciences, Shandong University, China (approval no.: SYDWLL-2023-023). The *Exosc10* Floxed mice were purchased from Cyagen, and the *Stra8-Cre* mouse line was generously provided by Professor Xiao-Yang Sun.

### Mouse genotyping

For genotyping, tail tissue samples were lysed using direct PCR lysis reagent containing proteinase K, with overnight incubation at 55 °C. The proteinase K was subsequently inactivated by incubating the samples at 85 °C for 1 h. PCR amplification of specific DNA fragments was performed using the PCR Master Mix (RiboBio Co, Ltd) and appropriate primers. The primer sequences used for PCR genotyping are listed in Table S1.

### Fertility assay

Male fertility was assessed by cohousing one wildtype female mouse with either a control or an *Exosc10* cKO male mouse for 6 months. The number of pups per litter was recorded, with at least three mating cages set up for each genotype.

### Histology analysis

Testes and epididymides from mice were fixed in Bouin’s solution at 4 °C overnight. After dehydration, the samples were embedded in paraffin, sectioned at 5 μm thickness, and stained with H&E. Bright field images were captured using a vertical microscope (Nexcope NE950).

### Immunofluorescence and TUNEL assay

For immunostaining, after dewaxing and rehydration, antigen retrieval was performed using 0.01% sodium citrate buffer (pH 6.0). Slides were blocked with a blocking buffer containing 0.05% Tween-20 at room temperature for 1 h, followed by overnight incubation with primary antibodies (Table S2) at 4 °C. The following day, slides were washed and incubated with secondary antibodies at 37 °C for 1 h. DNA was stained with Hoechst 33342. Apoptotic cells were analyzed using the TUNEL assay (Beyotime), following the manufacturer's instructions. Fluorescent images were captured using a fluorescent microscope (Nexcope NE950).

### RNA isolation and quantitative real-time RT–PCR

Total RNA was extracted from tissues or cells using the AFTSpin Tissue/Cell Fast RNA Extraction Kit for Animal (ABclonal, RK30120). Complementary DNA (cDNA) synthesis was carried out with the ABScript III RT Master Mix (ABclonal, RK20429). RT–qPCR was performed using the 2X Universal SYBR Green Fast qPCR Mix (ABclonal, RK21203). The primer sequences used in this study are provided in Table S3. The relative abundance of each transcript was calculated using the 2^−ΔΔ^Ct method, normalized to endogenous *β-actin* (35).

### Immunoblot assay

Total protein was extracted using 1x LDS sample buffer containing 1x NuPAGE sample reducing agent (Thermo Fisher Scientific). Proteins were separated on 4 to 12% Bis–Tris gels and transferred to polyvinylidene fluoride membranes. The membranes were blocked with 5% nonfat milk containing 0.05% Tween-20 for 1 h at room temperature and incubated with primary antibodies (Table S2) overnight at 4 °C. After washing the membranes three times with TBST, they were incubated with secondary antibodies (Table S2) at room temperature for 1 h. Signal detection was performed using SuperSignal West Dura Extended Duration Substrate (Thermo Fisher Scientific), and the signals were captured using a PXi system (Syngene) following the manufacturer's instructions. Uncropped blots are presented in Figure S10.

### PNA staining

For tissue staining, after dewaxing and rehydration, tissue sections were washed with PBS and stained with FITC-conjugated *Arachis hypogaea* PNA (15 μg/ml) at 37 °C for 1 h. The DNA was stained with Hoechst 33342, and images were captured using a fluorescent microscope (Nexcope NE950).

### Meiotic chromosome spreads

For germ cell isolation, after removal of the tunica albuginea, seminiferous tubules were incubated in hypotonic buffer (30 mM Tris [pH 7.5], 50 mM sucrose, 17 mM trisodium citrate, and 5 mM EDTA) for 30 min. The seminiferous tubules were then gently smashed to release germ cells into 200 mM sucrose. After treatment with fixation buffer (1% paraformaldehyde and 0.1% Triton X-100 in PBS), the cell suspensions were spread onto slides and air-dried.

### Enrichment of germ cells

For single-cell isolation, mouse testes were harvested and digested with collagenase IV (1 mg/ml). Dispersed seminiferous tubules were washed with D-Hanks solution and centrifuged at 200*g* for 5 min. The pellet was further digested with 0.25% trypsin containing DNase I (0.5 mg/ml) and filtered to prepare a single-cell suspension. The suspension was neutralized with 10% fetal bovine serum, filtered through a 70 μm cell strainer, and centrifuged again at 200*g* for 5 min at 4 °C. The cell pellet was resuspended in a complete medium (Dulbecco's modified Eagle's medium/F12 with 10% fetal bovine serum) and seeded in a 6-well cell culture plate. After culture at 37 °C in 5% CO_2_ for 16 h, the floating and weakly adherent cells were collected for subsequent experiments.

### scRNA-Seq library preparation

For scRNA-Seq, libraries were generated using Chromium Next GEM Single Cell 3′ Reagent Kits v3.1 (10× Genomics). Briefly, P21 single testicular cells from control and *Exosc10* cKO mice were mixed with a suspension containing barcoded beads and unique molecular identifier (UMI) elements. After partitioning thousands of cells into nanoliter-scale Gel Bead-In-Emulsions and barcoding, full-length barcoded cDNA was amplified by PCR for library construction. The libraries were constructed by fragmentation, end repair, A-tailing, adaptor ligation, and index PCR. cDNA libraries were sequenced on an Illumina NovaSeq 6000 platform at Guangzhou Gene Denovo Biotechnology Co, Ltd.

### scRNA-Seq data processing

Raw sequencing reads were aligned to the GRCm38 (mm10) reference genome and quantified using the CellRanger Software Suite (version 7.2.0; 10× Genomics). Raw gene expression matrices for each sample were generated using CellRanger, imported into R (version 4.2.0), and converted to a Seurat object using the Seurat R package (version 5.0.0) (36). Quality control was performed on the gene expression matrices, based on the distribution of detected genes, UMIs, and the percentage of mitochondrial UMIs. Cells with fewer than 200 or more than 7000 expressed genes, fewer than 2000 UMIs, or over 20% mitochondrial UMIs were filtered out.

### Single-cell transcriptomes to identify cell types

Normalization was applied to each sample using the Seurat R package, and the top 2000 highly variable genes were selected for principal component analysis dimensionality reduction. To correct for batch effects, the Harmony package (default parameters) was applied for integration (37). UMAP dimensionality reduction was then performed using the top 30 principal components. Cell clustering was conducted with a resolution of 1.2, and cell types were annotated based on known marker genes.

### Reclustering of the Scytes

For reclustering of Scytes, the top 2000 highly variable genes were selected for principal component analysis. Batch effect correction was applied using Harmony, followed by UMAP dimensionality reduction with the top 20 principal components. Clustering was performed at a resolution of 1.0, and cell types were identified based on classical marker genes.

### Identification of marker genes and DEGs

To identify marker genes in each cell type, gene expression levels in the cluster of interest were compared with those in other clusters using the Seurat *FindMarkers* function with the default Wilcoxon rank-sum test. DEGs between two samples were computed using the Seurat *FindMarkers* function, with parameters set to *p* < 0.05 and log_2_ fold change >0.2. GO analysis was conducted using Metascape to identify the biological processes and pathways (38).

### Pseudotime analysis

Pseudotime analysis was performed using Monocle 2 (39). The Seurat object for cell types was converted to a Monocle object, and data were reduced in dimensionality using the *reduceDimension* function with the DDRTree method, with results visualized accordingly.

### Statistical analysis

Data are presented as the mean ± SD. Statistical analyses were performed using a two-tailed Student’s *t* test, with significance defined as ns (no significance), ∗*p* < 0.05, ∗∗*p* < 0.01, ∗∗∗*p* < 0.001, and ∗∗∗∗*p* < 0.0001.

## Data availability

The raw sequencing data generated from this study have been deposited in the Sequence Read Archive with the accession number PRJNA1149533.

## Supporting information

This article contains supporting information.

## Conflict of interest

The authors declare that they have no conflicts of interest with the contents of this article.
