# Supplementary material for: Genetic deficiency of EXOSC10 ribonuclease disrupts spermatogenesis and male fertility in mice
Source: J Biol Chem. 2025 Jun 11;301(7):110364. doi: 10.1016/j.jbc.2025.110364 (PMC12269607; doi:10.1016/j.jbc.2025.110364)
Supplement: Supporting information [file mmc1.docx]

**
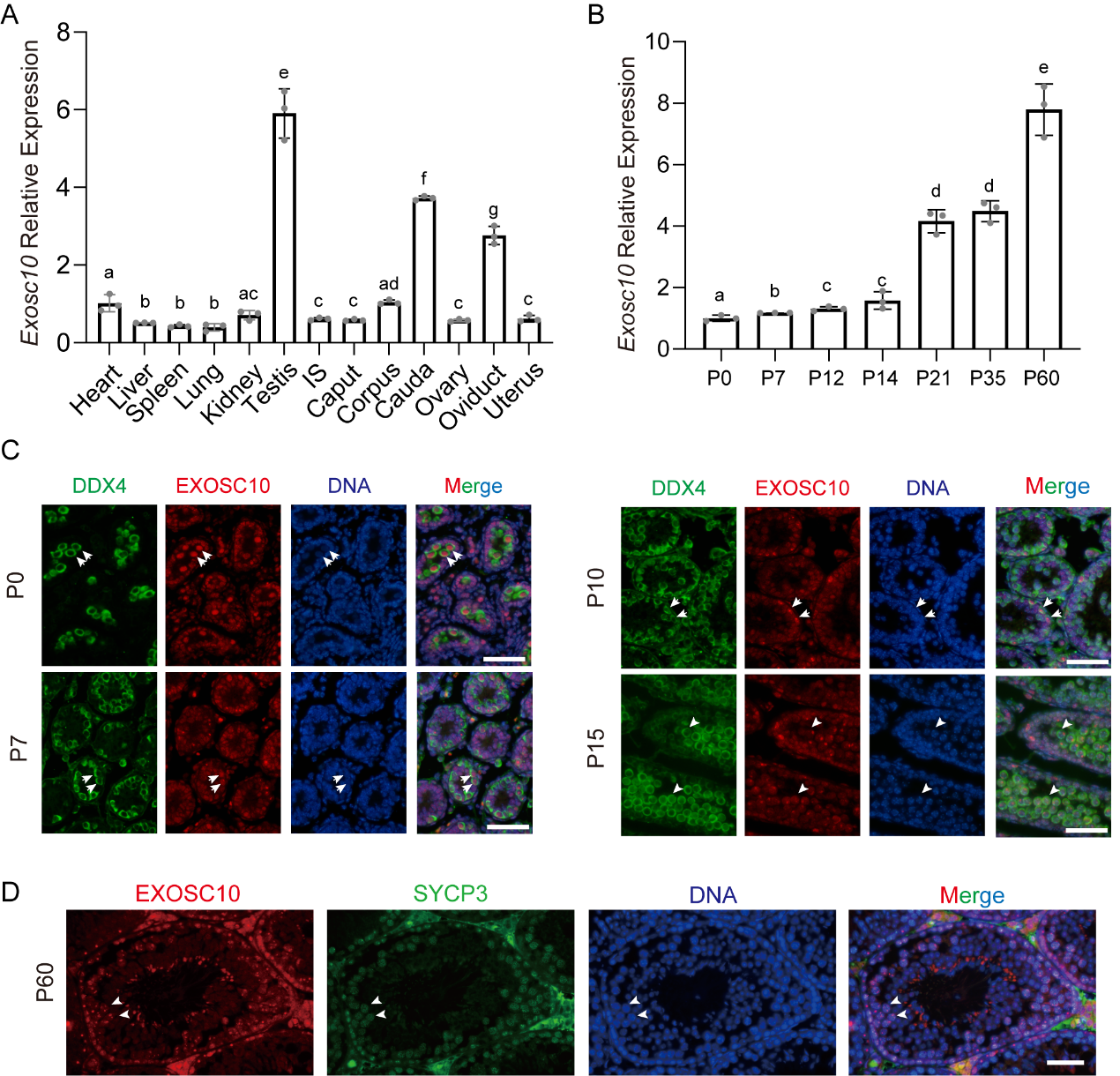
**

**Figure S1. EXOSC10 expression in mouse testes.** (A) RT-qPCR analysis of *Exosc10* expression in various organs of 2-month-old male mice. The expression level of *Exosc10* relative to *β-actin* in the heart was set to 1. Data are presented as mean ± s.d. from three biologically independent experiments. (B) Relative mRNA expression levels of *Exosc10* in male mouse testes at different ages. Expression at P0 is set to 1. Data are presented as mean ± s.d. from three biologically independent experiments. (C) Dual-immunofluorescence staining of DDX4 and EXOSC10 in testes at P0, P7, P10, and P15. Arrows and arrowheads indicate spermatogonia and spermatocytes, respectively. DNA was stained with Hoechst 33342. Scale bar, 50 μm. (D) Dual-immunofluorescence staining of EXOSC10 and SYCP3 in P60 wild-type testes. Arrowheads indicate spermatocytes. Scale bar, 50 μm. In multiple comparisons (A and B), the difference between different letters is significant (*P* < 0.05), while the difference between the same letters is not significant (*P* > 0.05).


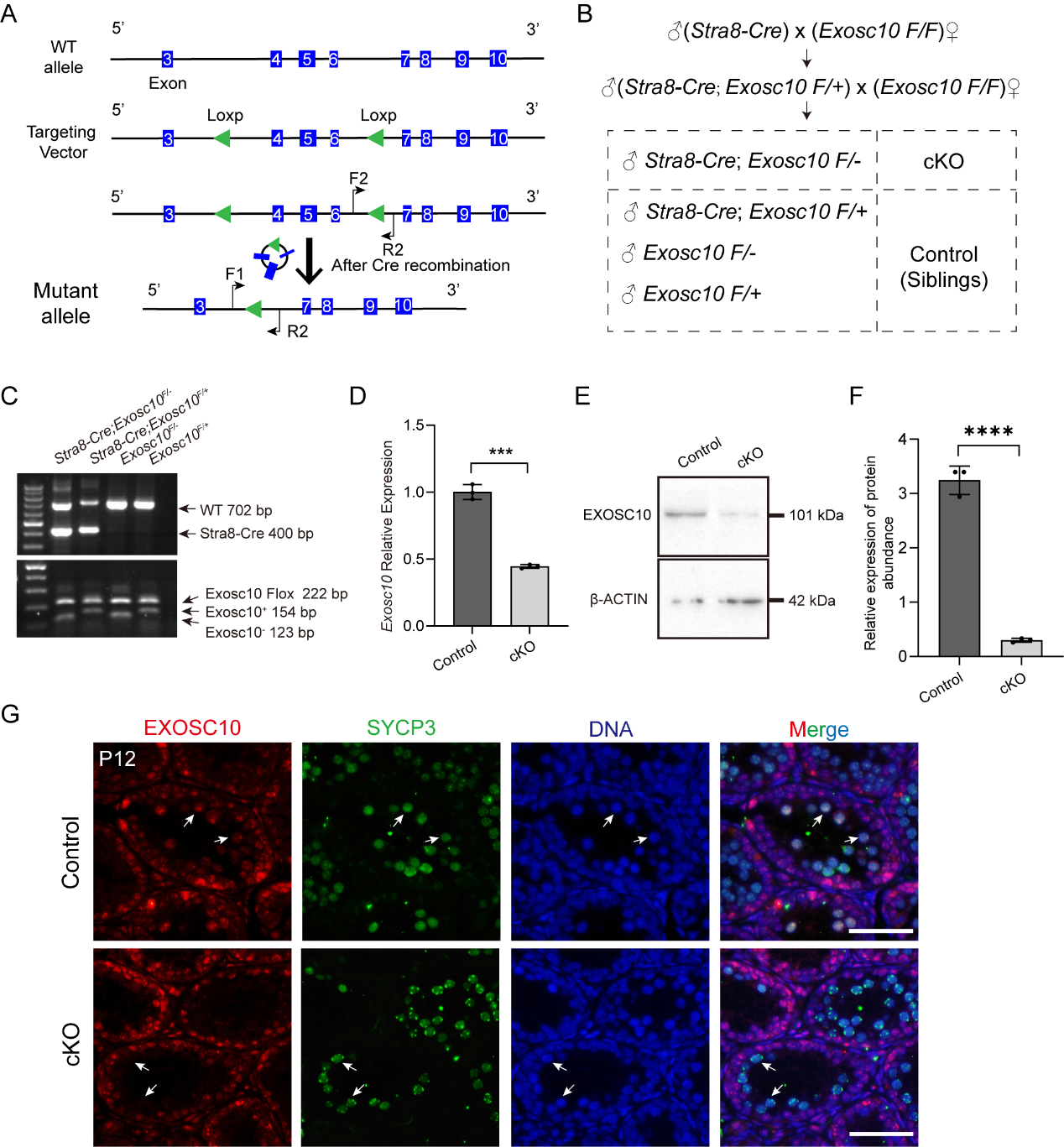


**Figure S2. Generation of** ***Exosc10* cKO mice.** (A) Schematic diagram of the *Exosc10* floxed allele, with exons 4-6 deleted after crossing with the *Stra8-Cre* line. Black arrows indicate primers used for genotyping. (B) Breeding scheme for generating *Exosc10* cKO mice. (C) Example of PCR results confirming the establishment of *Exosc10* cKO mice. (D) RT-qPCR analysis of *Exosc10* mRNA expression levels in P12 control and *Exosc10* cKO testes. The expression level of *Exosc10* relative to *β-actin* in control testes was set to 1. Data are presented as mean ± s.d. from three biologically independent experiments. ****P* < 0.001. (E) Western blot analysis of EXOSC10 protein levels in P12 control and *Exosc10* cKO testes. (F) Quantification of EXOSC10 protein levels in P12 control and *Exosc10* cKO testes. Data are presented as mean ± s.d. from three biologically independent experiments. *****P* < 0.0001. (G) Dual-immunofluorescence staining of EXOSC10 and SYCP3 in P12 control and *Exosc10* cKO testes. Arrows indicate SYCP3-positive spermatocytes lacking EXOSC10 signals. Scale bar, 50 μm.

**
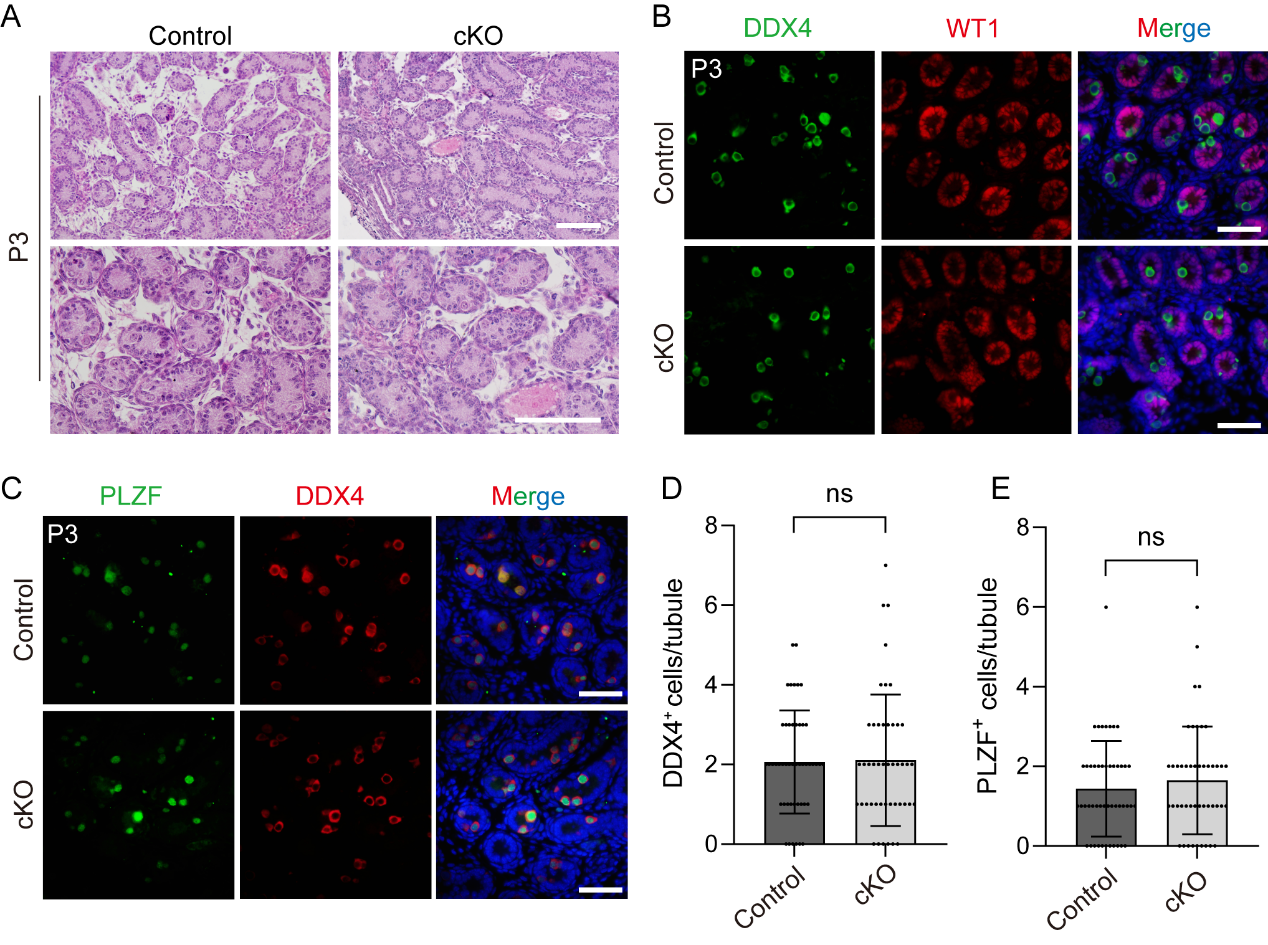
**

**Figure S3. Histological analysis of P3 *Exosc10* cKO testes.** (A) H&E staining of testes from P3 control and *Exosc10* cKO mice. Scale bar, 100 μm. (B) Dual-immunofluorescence staining of DDX4 and WT1 in testicular sections from P3 control and *Exosc10* cKO mice. DNA was stained with Hoechst 33342. Scale bar, 50 μm. (C) Dual-immunofluorescence staining of PLZF and DDX4 in testicular sections from P3 control and *Exosc10* cKO mice. Scale bar, 50 μm. (D, E) Statistical analysis of DDX4-positive (D) and PLZF-positive (E) cells per tubule in P3 control and *Exosc10* cKO testes. Data are expressed as mean ± s.d. from three biologically independent experiments; ns, no significance.


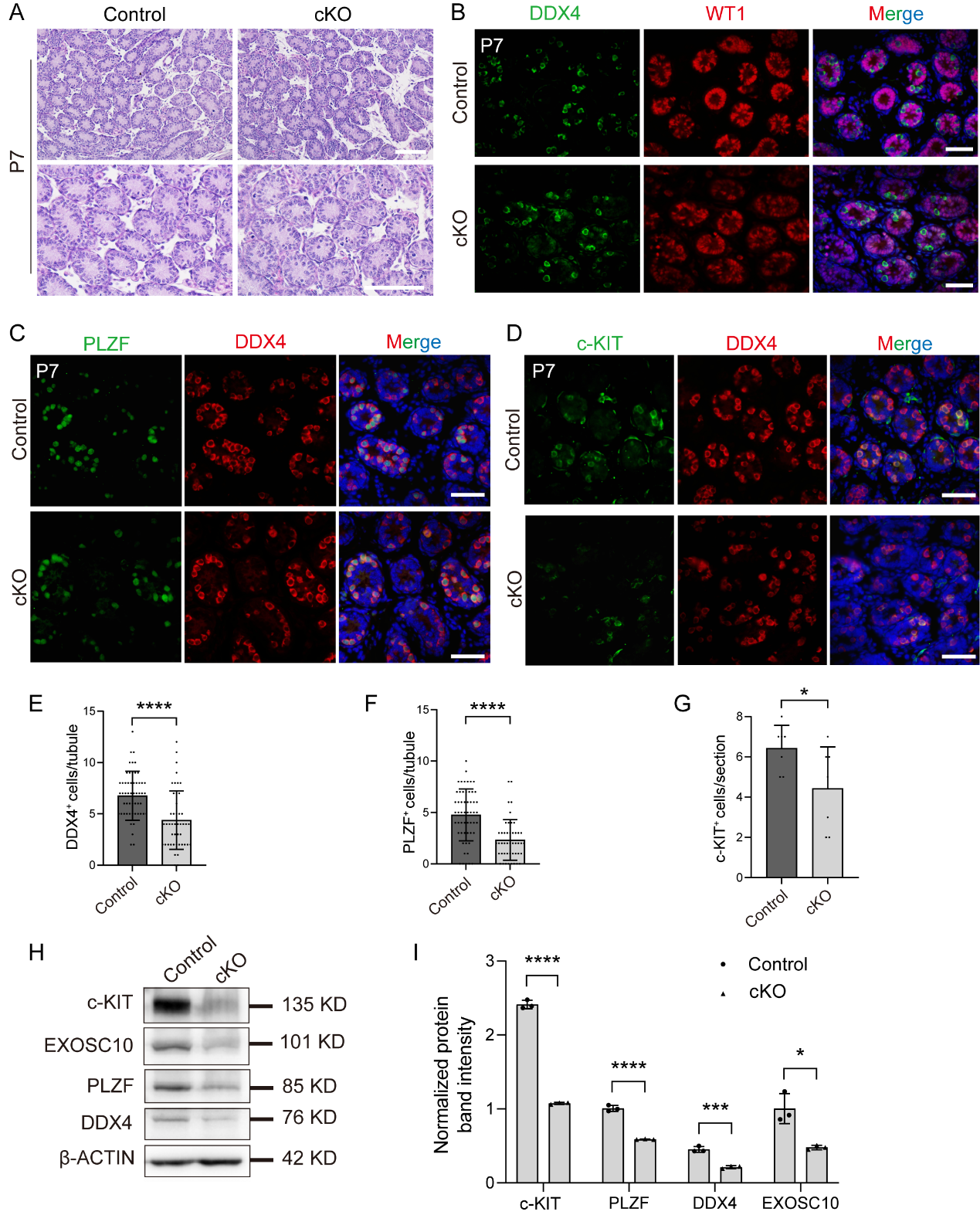


**Figure S4. Histological analysis of P7 *Exosc10* cKO testes.** (A) H&E staining of testes from P7 control and *Exosc10* cKO mice. Scale bar, 100 μm. (B) Dual-immunofluorescence staining of DDX4 and WT1 in testicular sections from P7 control and *Exosc10* cKO mice. Scale bar, 50 μm. (C) Dual-immunofluorescence staining of PLZF and DDX4 in testicular sections from P7 control and *Exosc10* cKO mice. Scale bar, 50 μm. (D) Co-immunostaining of c-KIT and DDX4 in testicular sections from P7 control and *Exosc10* cKO mice. Scale bar, 50 μm. (E-G) Quantification of DDX4-positive cells per seminiferous tubule (E), PLZF-positive cells per seminiferous tubule (F), and c-KIT-positive cells per section (G) in testes of P7 control and *Exosc10* cKO mice. Data are presented as mean ± s.d. from three biologically independent experiments; **P* < 0.05, *****P* < 0.0001. (H) Immunoblot assay of EXOSC10, PLZF, c-KIT, and DDX4 in P12 control and *Exosc10* cKO testes with β-ACTIN as a loading control. (I) Quantification of EXOSC10, PLZF, c-KIT, and DDX4 protein levels in P12 control and *Exosc10* cKO testes. Data are expressed as mean ± s.d. from three biologically independent experiments; **P* < 0.05, ****P* < 0.001, *****P* < 0.0001.


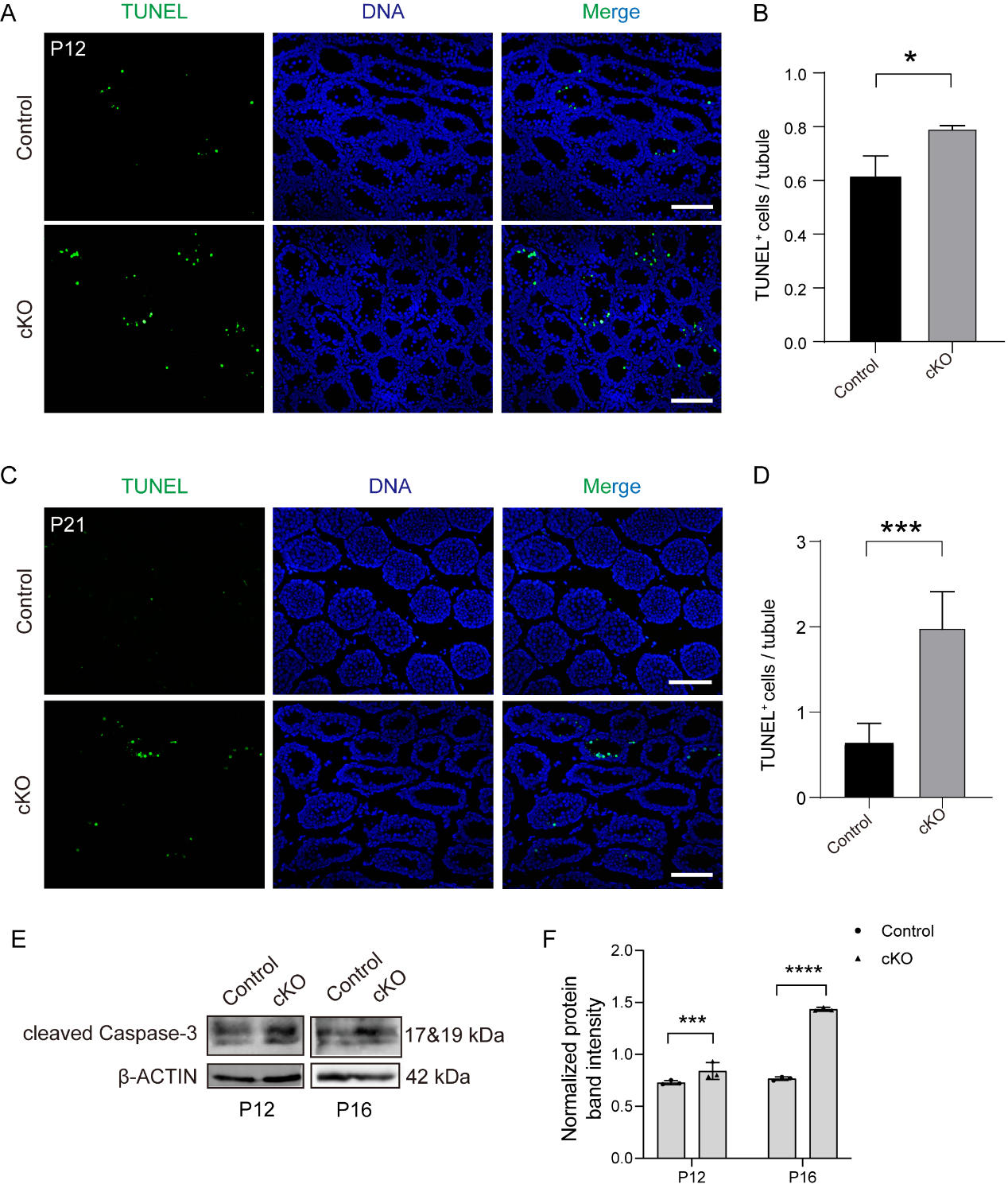


**Figure S5. Cell apoptosis in *Exosc10* cKO testes.** (A, C) TUNEL assay of testicular sections from control and *Exosc10* cKO mice at P12 (A) and P21 (C). Scale bar, 100 μm. (B, D) Quantification of TUNEL-positive cells per seminiferous tubule in P12 (B) and P21 (D) control and *Exosc10* cKO testes. Data are presented as mean ± s.d. from three biologically independent experiments; **P* < 0.05, ****P* < 0.001. (E) Immunoblot analysis of cleaved Caspase-3 in testes from P12 and P16 control and *Exosc10* cKO mice, with β-ACTIN as a loading control. (F) Quantification of cleaved Caspase-3 levels in testes from P12 and P16 control and *Exosc10* cKO mice. Data are expressed as mean ± s.d. from three biologically independent experiments; ****P* < 0.001, *****P* < 0.0001.


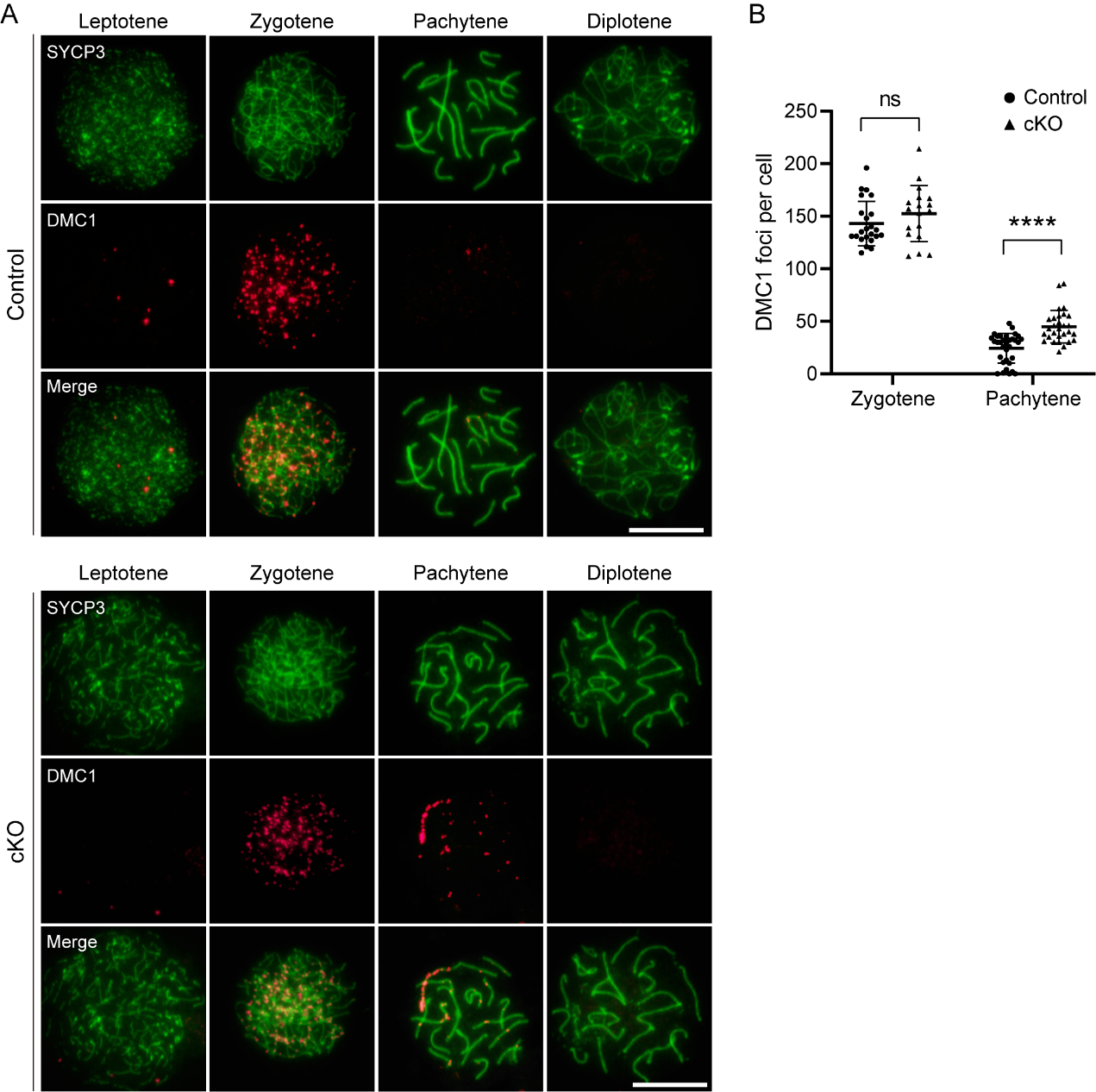


**Figure S6. Co-expression analysis of SYCP3 and DMC1 in *Exosc10* cKO testes.** (A) Chromosome spreads of spermatocytes from P16 control and *Exosc10* cKO mice were co-stained with antibodies against SYCP3 and DMC1. Scale bar, 10 μm. (B) Quantification of DMC1 foci during zygotene and pachytene stages in P21 control and *Exosc10* cKO mice. Data are expressed as mean ± s.d. from three biologically independent samples; ns, no significance; *****P* < 0.0001.


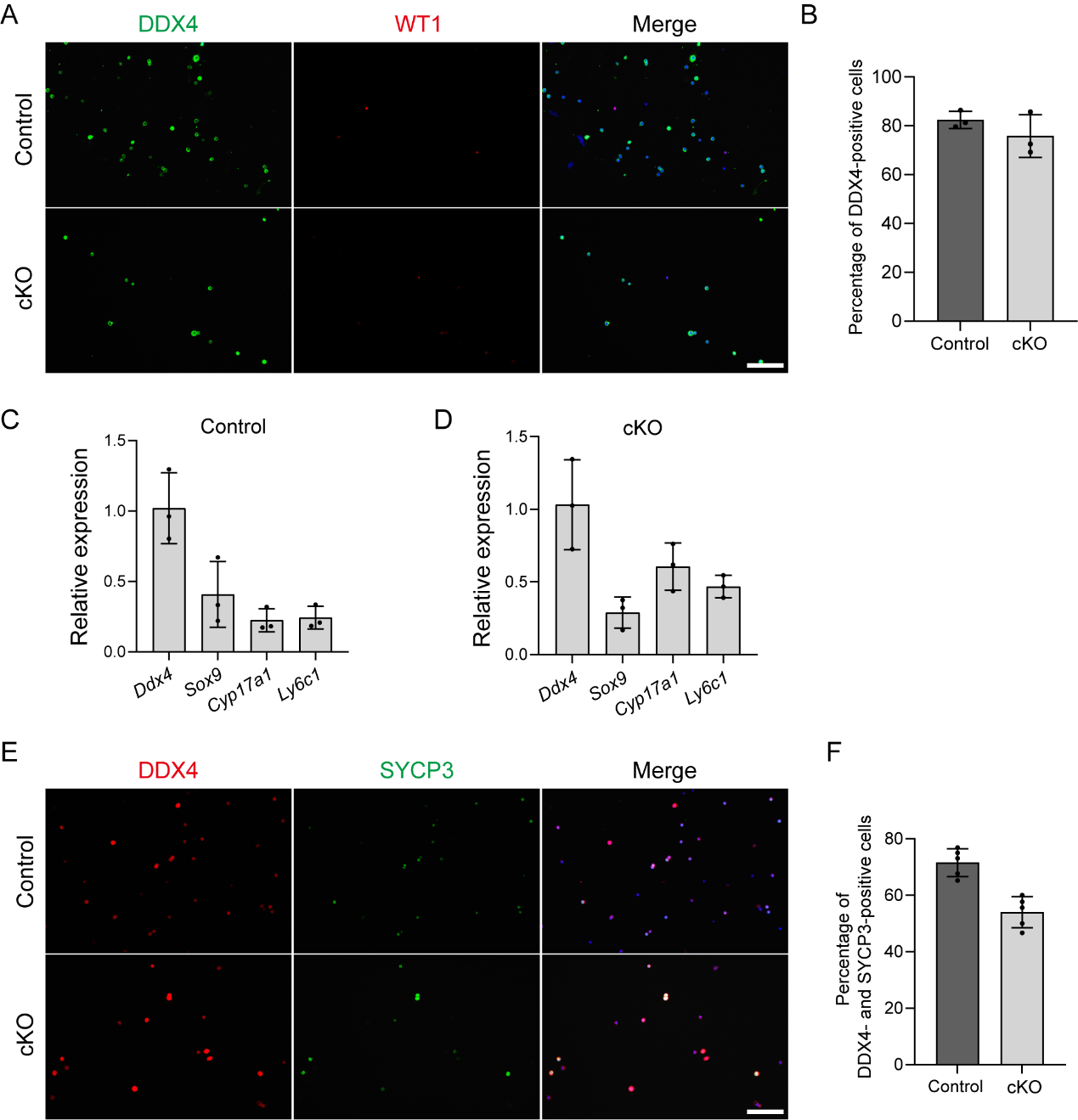


**Figure S7. Isolation of spermatogenic cells from P12 control and *Exosc10* cKO testes.** (A) Co-immunostaining of DDX4 and WT1 on isolated spermatogenic cells from control and *Exosc10* cKO mice. Scale bar, 100 μm. (B) Statistical analysis of the percentage of DDX4-positive cells. (C, D) RT-qPCR analysis of *Ddx4*, *Sox9*, *Cyp17a1*, and *Ly6c1* expression in isolated spermatogenic cells from control (C) and *Exosc10* cKO (D) mice, using *β-actin* as an internal control. The highest expression of *Ddx4* relative to *β-actin* was set to 1. (E) Co-expression of DDX4 and SYCP3 on isolated spermatogenic cells from control and *Exosc10* cKO mice. Scale bar, 100 μm. (F) Statistical analysis of the percentage of DDX4- and SYCP3-positive cells. Data are expressed as mean ± s.d. from three (B, C, D, and F) biologically independent replicates. Representative of three (A and E) biologically independent replicates with similar results per condition.


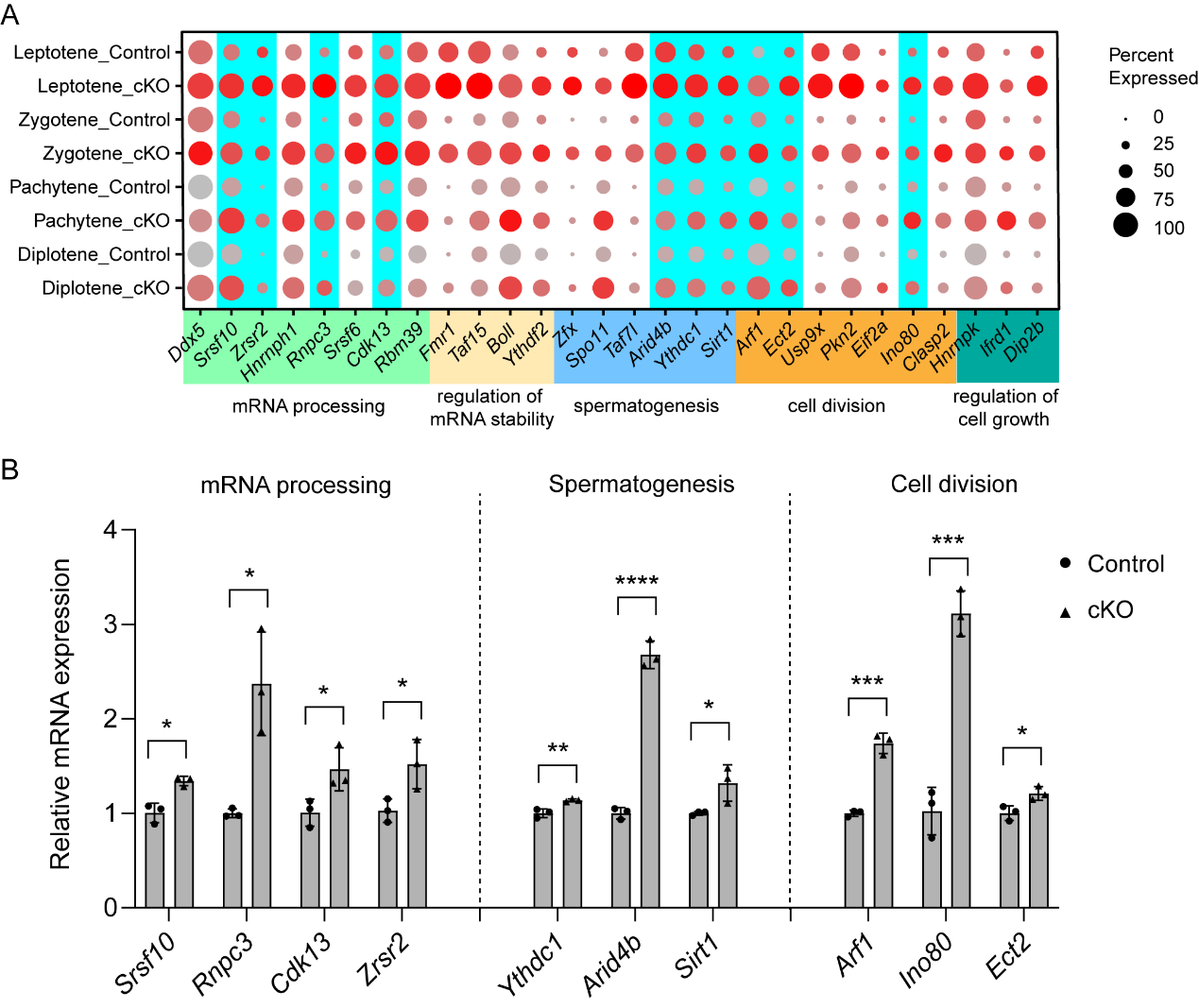


**Figure S8. Differential transcript upregulation from leptotene to diplotene in *Exosc10* cKO spermatocytes.** (A) Dot plot displaying selected upregulated genes associated with mRNA processing, regulation of mRNA stability, spermatogenesis, cell division, and regulation of cell growth. (B) RT-qPCR analysis of expression levels of genes linked to mRNA processing, spermatogenesis, and cell division in isolated spermatogenic cells from control and *Exosc10* cKO mice. The expression level of genes in control relative to *β-actin* was set to 1. Data are presented as mean ± s.d. from three biologically independent replicates. **P* < 0.05, ***P* < 0.01, ****P* < 0.001, and *****P* < 0.0001.


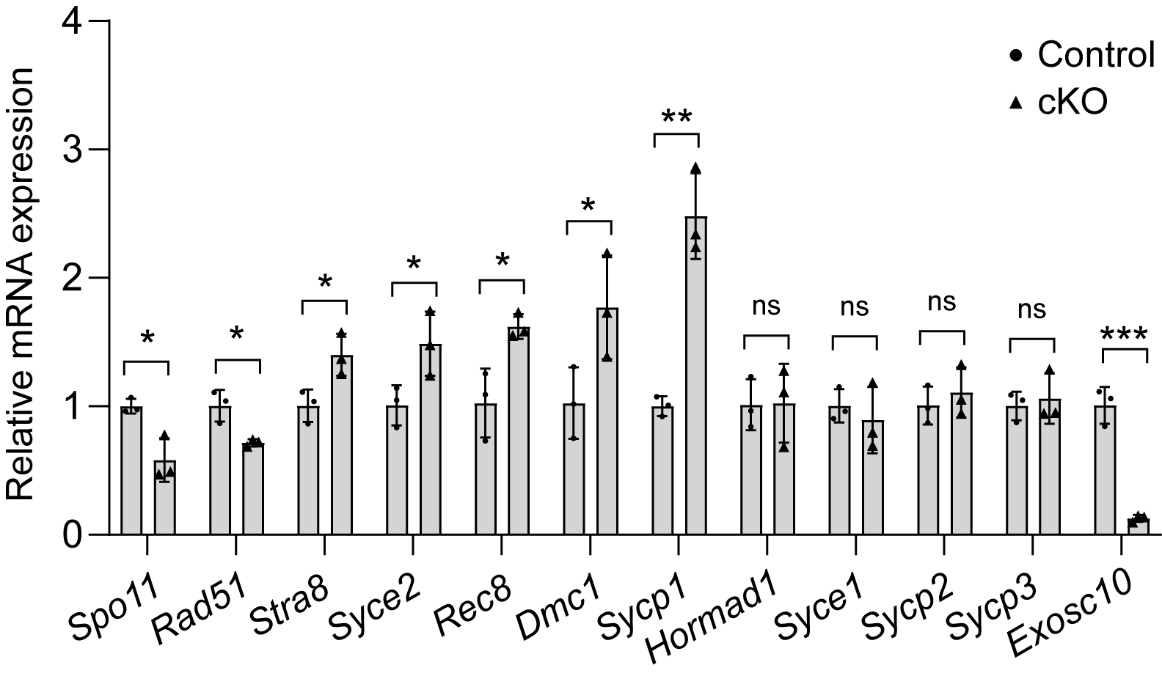


**Figure S9. Dysregulation of meiotic genes in *Exosc10* cKO germ cells.** RT-qPCR analysis of meiotic gene expression in isolated spermatogenic cells from control and *Exosc10* cKO mice. Data are presented as mean ± s.d. from three biologically independent replicates. The expression levels of genes in control relative to *β-actin* were set to 1. Ns, no significance, **P* < 0.05, ***P* < 0.01, ****P* < 0.001.


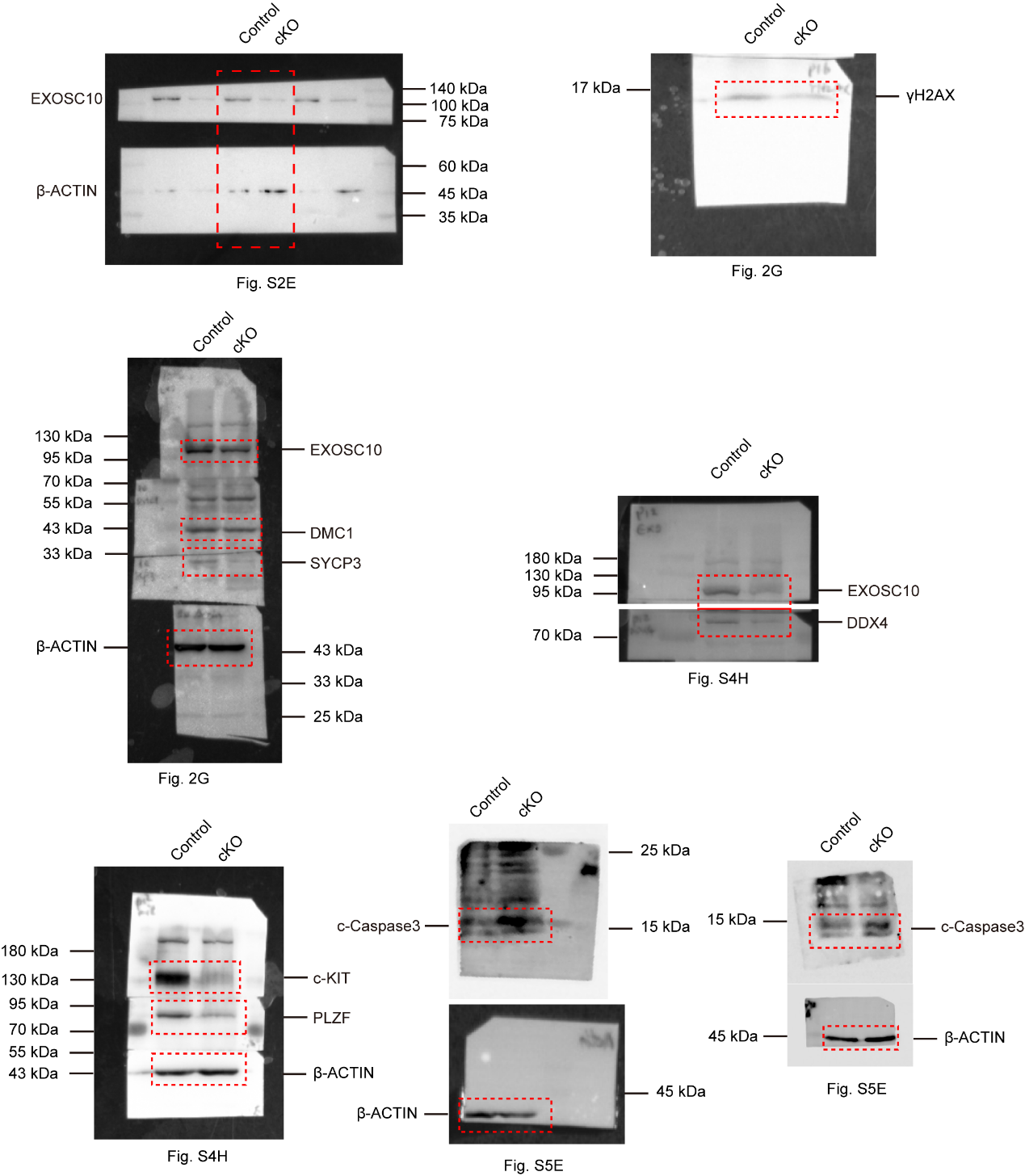


**Figure S10. Uncropped immunoblot images in this study.** Uncropped immunoblot images for Fig. 2G, Fig. S2E, Fig. S4H and Fig. S5E.

**Table S1. Genotyping primers for *Exosc10* cKO and *Stra8*-Cre mice**

| Gene | Direction | Primer (5’-3’) |
| --- | --- | --- |
| *Stra8*-Cre  (Internal positive control) | F^1^ | ACTCCAAGCACTGGGCAGAA |
|  | R | GCCACCATAGCAGCATCAAA |
| *Stra8*-Cre | F | ACTCCAAGCACTGGGCAGAA |
|  | R | CGTTTACGTCGCCGTCCAG |
| *Exosc10* Flox | F | AAGTAATTCTGAGCTGTCTGCCA |
|  | R | CCGGGCAGTACATATACCCAAAG |
| *Exosc10* Deletion | F | ACGTAAACGGCCACAAGTTC |
|  | R | CCGGGCAGTACATATACCCAAAG |

^1^F, forward; R, reverse

**Table S2. Antibodies used in this study**

| Antibody | Company | Identifier | Immunohisto-chemistry | Immunoblot |
| --- | --- | --- | --- | --- |
| Mouse anti-DDX4 | Abcam | Cat# ab27591;  RRID: AB_11139638 | 1:200 |  |
| Rabbit anti-DDX4 | Abcam | Cat# ab13840;  RRID: AB_443012 | 1:200 | 1:1000 |
| Rabbit anti-WT1 | Abcam | Cat# ab89901;  RRID: AB_2043201 | 1:200 |  |
| Mouse anti-SYCP3 | Abcam | Cat# ab97672  RRID: AB_10678841 | 1:200 | 1:1000 |
| Goat anti-PLZF | R&D Systems | Cat# AF2944;  RRID: AB_2218943 | 1:200 | 1:1000 |
| Rabbit anti-MLH1 | Proteintech | Cat# 11697-1-AP | 1:150 |  |
| Rabbit anti-EXOSC10 | Abcam | Cat# ab50558 | 1:200 | 1:1000 |
| Rabbit anti-SYCP1 | Abcam | Cat# ab15090 | 1:200 |  |
| Rabbit anti-DMC1 | Proteintech | Cat# 13714-1-AP | 1:200 | 1:1000 |
| Rabbit anti-Actin | ABclonal | Cat# AC026 |  | 1:10000 |
| Rabbit anti- cleaved Caspase-3 | Affinity | Cat# AF7022 |  | 1:1000 |
| Goat anti-KIT | R&D Systems | Cat# AF1356;  RRID: AB_354750 | 1:200 | 1:800 |
| Rabbit anti-phospho-histone H2A.X (Ser139) | Cell Signaling Technology | Cat# 9718;  RRID: AB_2118009 | 1:200 | 1:1000 |
| Donkey anti-mouse IgG, Alexa Fluor 488 | Thermo Fisher Scientific | Cat# A-21202 | 1:200 |  |
| Donkey anti-rabbit IgG, Alexa Fluor 594 | Thermo Fisher Scientific | Cat# A-21207 | 1:200 |  |
| Donkey anti-goat IgG, Alexa Fluor 488 | Thermo Fisher Scientific | Cat# A-11055 | 1:200 |  |
| Donkey anti-mouse IgG, Alexa Fluor 594 | Thermo Fisher Scientific | Cat# A-21203 | 1:200 |  |
| Goat anti-mouse IgG, HRP | Thermo Fisher Scientific | Cat# 62-6520 |  | 1:5000 |
| Goat anti-rabbit IgG, HRP | Thermo Fisher Scientific | Cat# 31460 |  | 1:5000 |
| Donkey anti-Goat IgG, HRP | Thermo Fisher Scientific | Cat# PA1-28664 |  | 1:5000 |

**Table S3. Primers used for RT-PCR**

| Gene | Direction | Primer (5’-3’) |
| --- | --- | --- |
| *Exosc10* | F^1^ | AGAGAGTGGGCATGTTACTGG |
|  | R | GCTGAGGTCGCACGATGTT |
| 5’ETS rRNA | F | TGTTTCACTTTGGTCGTGTCTC |
|  | R | TCGACGCTTACAAGAAACAGC |
| 47S rRNA | F | GGTGTCCAAGTGTTCATG |
|  | R | CAAGCGAGATAGGAATGTCTTAC |
| 18S rRNA | F | TGATTAAGTCCCTGCCCTTTG |
|  | R | CTTCTCTCACCTCACTCCAGACAC |
| ITS1 rRNA | F | TCTCGTTTCGTTCCTGCTGG |
|  | R | GATCCACCGCTAAGAGTCGTATC |
| ITS2 rRNA | F | CGTGTGAGTAAGATCCTCCAC |
|  | R | GTTACTGAGGGAATCCTGGTTAG |
| 28S rRNA | F | CGTGTGAGTAAGATCCTCCACC |
|  | R | GAGTTTACCACCCGCTTTGG |
| *Rps6* | F | AGCTCCGCACCTTCTATGAGA |
|  | R | GGGAAAACCTTGCTTGTCATTC |
| *Rpl11* | F | ATGGCGCAAGATCAAGGGG |
|  | R | GACTGTGCAGTGAACAGCAAT |
| *Fau* | F | GCCCAGGAACTACACACCC |
|  | R | AAGCACGACTTGATCTTCGGG |
| *Rpl35a* | F | TGTGGTGCAAGGCCATTTTTG |
|  | R | CTCCGTTTCATCTCGGGCATA |
| *Rps7* | F | AGCGCCAAGATCGTGAAGC |
|  | R | CACCACCAACTTCGATTTCCTT |
| *Rpl7* | F | ACCGCACTGAGATTCGGATG |
|  | R | GAACCTTACGAACCTTTGGGC |
| *Rpl26* | F | ACTTCTGACCGAAGCAAGAAC |
|  | R | CCGAATGGGCATAGACCGAA |
| *Rnf151* | F | TGCAAGTTCCTGTGTTCTGTC |
|  | R | CCTTGTCACCTCTTTTCTACAGC |
| *Gpx4* | F | GATGGAGCCCATTCCTGAACC |
|  | R | CCCTGTACTTATCCAGGCAGA |
| *Odf2* | F | GCCAGGTGGAGTCGAATCTG |
|  | R | GTTCGGCTGACGTGAAGGAG |
| *Ggnbp1* | F | CCTCGGTCACGGATCTTAGG |
|  | R | CCCATCATTGGGGAGACAACAT |
| *Rps16* | F | CACTGCAAACGGGGAAATGG |
|  | R | CACCAGCAAATCGCTCCTTG |
| *Stra8* | F | CAAAAGCCTTGGCTGTGTTA |
|  | R | AAAGGTCTCCAGGCACTTCA |
| *Sycp1* | F | CAAAAGCCCTTCACACTGTTCG |
|  | R | GTTTTCCCGACTGGACATTGTAA |
| *Sycp2* | F | AGGATGAGATCACTACACCTAGC |
|  | R | GGTGACGCAGCATAATCCATT |
| *Sycp3* | F | AGCCAGTAACCAGAAAATTGAGC |
|  | R | CCACTGCTGCAACACATTCATA |
| *Syce1* | F | GCATGTTGCAGGAGTGTAAAGA |
|  | R | GCTGCTGTCCAAAACACACATC |
| *Syce2* | F | TGGACTCTAGCATTGAAACCCT |
|  | R | TCCTGAATGATTTTGCTGTGGT |
| *Rec8* | F | TATGTGCTGGTAAGAGTGCAAC |
|  | R | TGTCTTCCACAAGGTACTGGC |
| *Spo11* | F | CGTGGCCTCTAGTTCTGAGGT |
|  | R | GCTCGATCTGTTGTCTATTGTGA |
| *Dmc1* | F | CCCTCTGTGTGACAGCTCAAC |
|  | R | GGTCAGCAATGTCCCGAAG |
| *Rad51* | F | AAGTTTTGGTCCACAGCCTATTT |
|  | R | CGGTGCATAAGCAACAGCC |
| *Hormad1* | F | GGCTCCTAGCTGTTTCAGTATCT |
|  | R | TTGTCCCATAAGCACGTTCTG |
| *β-actin* | F | GGCTGTATTCCCCTCCATCG |
|  | R | CCAGTTGGTAACAATGCCATGT |

^1^F, forward; R, reverse
